# Galactooligosaccharides Attenuate Behavioural, Haematological and Immunological Abnormalities and Influence Gut Microbiota in Rats with Amygdala Hyperactivation Induced by Electrical Stimulation

**DOI:** 10.3390/ijms26094353

**Published:** 2025-05-03

**Authors:** Jan Ruciński, Ewelina Kurowska-Rucińska, Dorota Myślińska, Beata Grembecka, Natalia Piekarczyk, Agnieszka Necel, Katarzyna Kosznik-Kwaśnicka, Irena Majkutewicz

**Affiliations:** 1 Department of Animal and Human Physiology, Faculty of Biology, University of Gdańsk, Wita Stwosza 59 St., 80-308 Gdańsk, Poland; jan.rucinski@ug.edu.pl (J.R.); ewelina.kurowska@ug.edu.pl (E.K.-R.); dorota.myslinska@ug.edu.pl (D.M.); beata.grembecka@ug.edu.pl (B.G.); natalia.piekarczyk@ug.edu.pl (N.P.); 2 Division of Medical Microbiology, Department of Microbiology, Faculty of Medicine, Medical University of Gdańsk, M. Skłodowskiej-Curie 3a St., 80-210 Gdańsk, Poland; agnieszka.necel@gumed.edu.pl (A.N.); katarzyna.kwasnicka@gumed.edu.pl (K.K.-K.)

**Keywords:** amygdala, galactooligosaccharides, prebiotics, gut microbiota, dysbiosis, behaviour, stress response, inflammation, neuropsychiatric disorders

## Abstract

The amygdala, especially its central nucleus (CeA), is one of the key brain structures regulating fear, anxiety and stress responses and is also involved in gut microbiota signal processing. Amygdala hyperactivity, as well as microbiota alterations, plays an important role in the pathophysiology of anxiety disorders, depression or post-traumatic stress disorder (PTSD). The present study determines whether 3 weeks of galactooligosaccharide (GOS) supplementation alleviates behavioural, haematological, immunological and gut microbiota disturbances induced by long-term electrical stimulation of the CeA in rats (Stim). The unsupplemented Stim group showed locomotor hyperactivity and higher anxiety (measured with an actometer and the elevated plus maze, respectively), as well as a decrease in white blood cells (WBCs), lymphocytes (LYMs), red blood cells (RBCs) and platelets (PLTs); an elevation of TNFα; a reduction in IL-10 concentration in plasma; and microbiota alterations as compared to the control (Sham) group. GOS supplementation alleviated all these Stim-induced adverse effects or even normalised them to the sham group level. The effect of GOS was comparable to citalopram and even more effective in WBC and PLT normalisation and IL-10 induction. The obtained results indicate the high therapeutic potential of GOS in anxiety and stress-related disorders. GOS supplementation may support conventional therapy or the prevention of PTSD, depression and anxiety disorders.

## 1. Introduction

Neuropsychiatric disorders associated with anxiety and stress such as depression, anxiety disorders or post-traumatic stress disorder (PTSD) are currently a significant health, social and economic problem. About 40% of the population suffers from these types of disorders, and the number of diagnosed patients has increased over the last few decades. The symptoms of these diseases affect the health of patients, the ability to maintain a normal lifestyle and social skills, including working or studying. The most frequently mentioned etiological factors include genetic predisposition, chronic abnormalities in the stress response and long-term inflammation, as well as gut microbiota dysfunctions [1,2,3]. The amygdala (Amg), especially its central nucleus (CeA), is one of the key structures of the central nervous system (CNS) regulating reactions related to fear, anxiety and the stress response and is also involved in gut microbiota signal processing [4,5,6]. It has been proven that Amg hyperactivity plays an important role in the pathophysiology of anxiety disorders, depression and PTSD, mainly through the strong influence of this structure on the neuroendocrine, autonomic and behavioural components of the stress response [7,8,9,10]. The mechanisms associated with Amg hyperactivation in the development and course of the above-mentioned diseases are not fully understood. Moreover, research on this issue most often uses animal models of acute or chronic stress, in which there is nonspecific modulation of the activity of many areas of the CNS, including Amg. The rat model of electrical stimulation of the CeA used in this study was intended to induce symptoms resembling those occurring in patients suffering from anxiety disorders, depression or PTSD directly induced by long-term Amg hyperactivation [11,12,13,14,15]. Therefore, the first aim of this study was to verify that the hyperactivity of the Amg evoked by long-term electrical stimulation of the CeA induces haematological, immunological and behavioural abnormalities, as well as causes changes in the rats’ gut microbiota.

Many studies have demonstrated the presence of functional bidirectional communication between the gut microbiota and the CNS via the gut–brain axis. Intestinal microorganisms and their neuroactive metabolites can modulate immune and stress responses, as well as CNS functions, including behaviour, mood, emotions or cognitive processes [16,17,18]. The growing body of evidence confirms a link between gut microbiota dysbiosis and neuropsychiatric disorders. Qualitative and quantitative disturbances in the gut microbiota composition occur in patients with depression, anxiety disorders and PTSD, affecting the severity of changes related to inflammation and hypothalamic–pituitary–adrenal (HPA) axis activity and leading to CNS dysfunction. It was also confirmed that both acute and chronic stress can induce gut dysbiosis [19,20,21,22]. These reports have contributed to the development of research using probiotics and prebiotics as agents capable of modulating the gut microbiota composition [23,24,25,26]. Galactooligosaccharides (GOS) are indigestible complex sugar oligosaccharides, which, unlike probiotics, promote the proliferation and activity of native beneficial bacteria species in the host gastrointestinal tract [27,28]. The effect of GOS on CNS function in the context of neuropsychiatric disorders has been assessed in several preclinical and clinical studies, most of which confirmed their beneficial effects [29,30,31,32,33,34], although there are also reports in which no significant changes caused by GOS supplementation have been noted [35,36,37]. Further studies on the GOS therapeutic potential seem to be necessary; therefore, the next aim of our study was to determine the effect of GOS supplementation on behavioural, haematological and immunological changes induced by long-term electrical stimulation of the CeA in rats. Additionally, the present study aims to compare the effectiveness of GOS supplementation with the effects of citalopram (CIT) therapy, an anxiolytic and antidepressant drug, which is commonly used in patients with depression and anxiety disorders [38].

## 2. Results

### 2.1. Locomotor Activity

**Figure 1:**
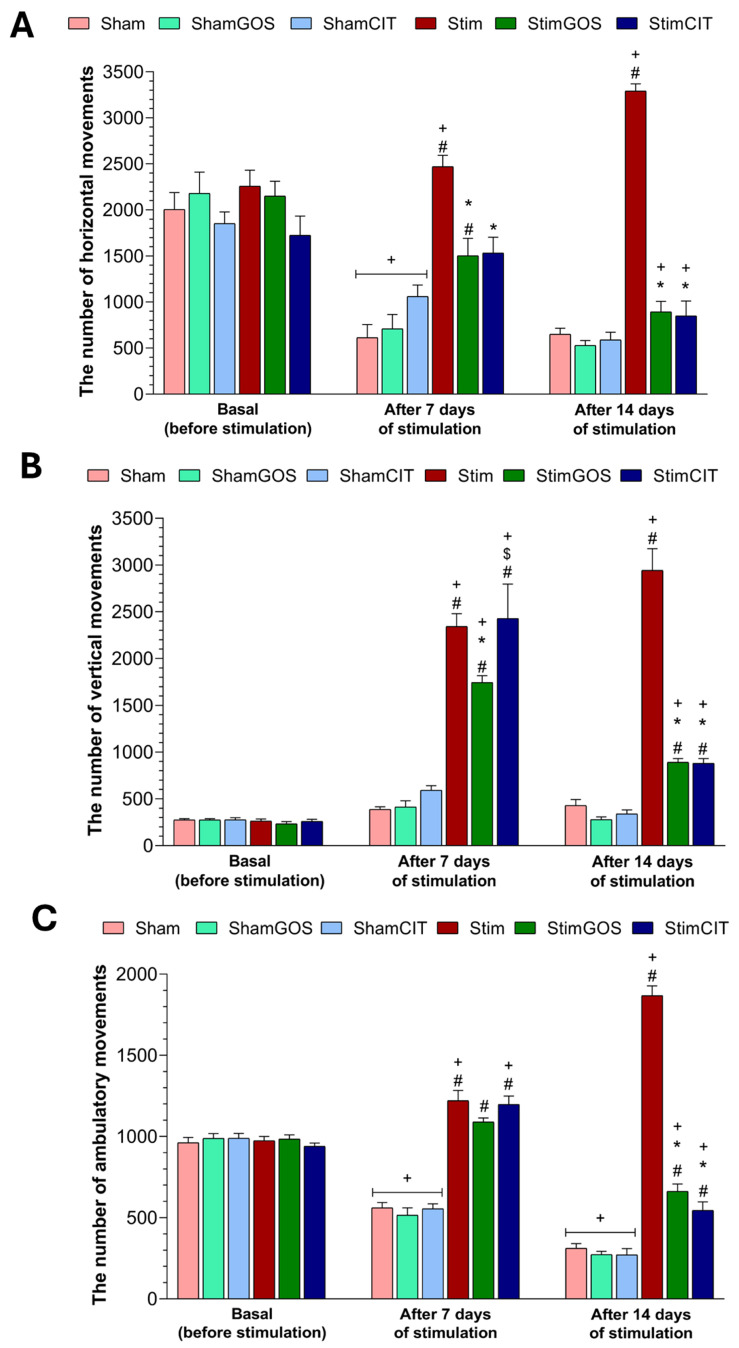
Effects of GOS supplementation or CIT administration on locomotor activity expressed as the number of horizontal (**A**), vertical (**B**) and ambulatory (**C**) movements in the actometer at three time points: basal (before stimulation), after 7 days and after 14 days of stimulation in rats subjected to long-term electrical stimulation of the CeA or sham stimulation. Data are expressed as the mean ± SEM; n = 8 animals/group. Explanations: # *p* < 0.05 stimulated vs. sham rats within the same supplementation group (Stim vs. Sham; StimGOS vs. ShamGOS; StimCIT vs. ShamCIT); * *p* < 0.05 certain stimulated and supplemented groups vs. Stim; $ *p* < 0.05 StimGOS vs. StimCIT; + *p* < 0.05 the certain time point vs. previous within the same experimental group using three-way ANOVA followed by Tukey’s post hoc test.

Increased animal motor activity during electrical stimulation, especially in the vertical and horizontal planes, may indicate a desire to escape from an unpleasant environment or test conditions. Initially, in the novelty test, in which the actometer is a new environment for the animal, the basal level of horizontal (HOR), vertical (VER) and ambulatory (AMB) movements was assessed (Figure 1). Then, the rats’ locomotor activity was recorded during each day of the CeA electrical stimulation or sham stimulation. To analyse the effect of electrical stimulation and the administration of GOS or CIT, the mean number of each type of recorded movement (HOR, VER and AMB) was calculated for the following two time points: (1) after 7 days of stimulation (days 1–7) and (2) after 14 days of stimulation (days 8–14) (Figure 1). The three-way ANOVA revealed that all tested factors, namely stimulation (stimulated_sham), supplementation (GOS_CIT_water) and time point (basal_after 7 days_after 14 days of stimulation), significantly influenced the number of each type of movement (HOR, VER and AMB). Interactions between those factors were also significant (Table 1).

Before stimulation (time point: basal), during the novelty test, rats from each experimental group were characterised by a similar number of horizontal (Figure 1A), vertical (Figure 1B) and ambulatory (Figure 1C) movements, and the observed differences were not statistically significant.

Sham, ShamGOS and ShamCIT animals showed a significant reduction in the number of horizontal (*p* < 0.001 in each sham group) and ambulatory movements (*p* < 0.001 in each sham group) and a non-significant increase in the number of vertical movements after 7 days of sham stimulation in relation to the basal results. In the last time point, the number of horizontal and vertical movements were similar to the results obtained in the previous stage (no significant changes after 14 days vs. 7 days of the stimulation procedure), but the number of ambulatory movements decreased significantly in all sham groups (Sham: *p* = 0.001, ShamGOS: *p* = 0.002 and ShamCIT: *p* < 0.001). There was also no significant effect of GOS or CIT on the number of each type of movement in rodents subjected to sham stimulation at any stage of the experiment. This may indicate a gradual adaptation of the animals to the environment and test conditions.

The electrical stimulation of the CeA after just 7 days caused significant changes in the locomotor activity of stimulated and unsupplemented rats (Stim group). Animals from the Stim group showed a statistically significant higher number of movements in the horizontal and vertical planes, and the number of ambulatory movements in this time point in relation to basal results (HOR: *p* = 0.04, VER: *p* < 0.001 and AMB: *p* = 0.002), as well as compared to rodents subjected to sham stimulation without supplementation (Sham group) (*p* < 0.001 in each type of movement), was significant. This effect was intensified after the next 7 days, which resulted in a higher level of locomotor activity in each type of movement in Stim rats after 14 days of electrical stimulation of the CeA compared to the result from the previous time point (HOR: *p* = 0.013, VER: *p* = 0.023 and AMB: *p* < 0.001) and compared to the number of movements of the Sham group (*p* < 0.001 in each type of movement).

GOS supplementation significantly reduced the level of locomotor activity (horizontal and vertical movements) induced by electrical stimulation of the CeA after just 7 days of stimulation in StimGOS rats, which was after two weeks of supplementation (StimGOS vs. Stim HOR: *p* < 0.001 and VER: *p* = 0.024). At this time point of the experiment, only the number of vertical movements was statistically significantly higher in the StimGOS group in relation to basal results (*p* < 0.001). After 14 days of stimulation (21 days of GOS supplementation) the difference between the StimGOS and Stim animals was even greater (*p* < 0.001 in each type of movement), whereas the number of each type of movement within the StimGOS group was significantly reduced at this time point of the experiment compared to the previous stage (after 14 days vs. 7 days of the stimulation procedure, HOR: *p* = 0.033, VER: *p* < 0.001 and AMB: *p* < 0.001). Compared to the sham-stimulated and GOS-supplemented rats, StimGOS animals showed a statistically significant higher number of horizontal movements after 7 days of the stimulation procedure (*p* = 0.021); however, after 14 days, GOS administration to stimulated animals led to the complete normalisation of motor activity in the horizontal plane (no statistically significant differences between the StimGOS and ShamGOS groups). The number of vertical and ambulatory movements was significantly higher in the second (*p* < 0.001 in each type of movement) and third (VER: *p* = 0.017 and AMB: *p* < 0.001) time points of experiment in StimGOS rats compared to the ShamGOS group.

Rats with CIT therapy showed a similar pattern of changes in locomotor activity to StimGOS animals when compared to the Stim group. However, CIT administration to the stimulated rats did not cause significant differences in vertical motor activity between the StimCIT and Stim groups after 7 days of stimulation, and the number of vertical movements in the StimCIT group was statistically significantly higher compared to the StimGOS group (*p* = 0.003). After 14 days of stimulation, the difference between these two groups was eliminated; stimulated and supplemented rodents showed a similar level of motor activity in the vertical plane, which is also significantly lower compared to Stim rats (*p* < 0.001). Similarly to GOS supplementation, CIT administration did not contribute to the complete normalisation of vertical movements of animals stimulated and receiving CIT, in which the number of these movements was statistically significantly higher compared to the ShamCIT group after 7 (*p* < 0.001) and 14 days of stimulation (*p* = 0.047).

### 2.2. The Anxiety Level

**Figure 2:**
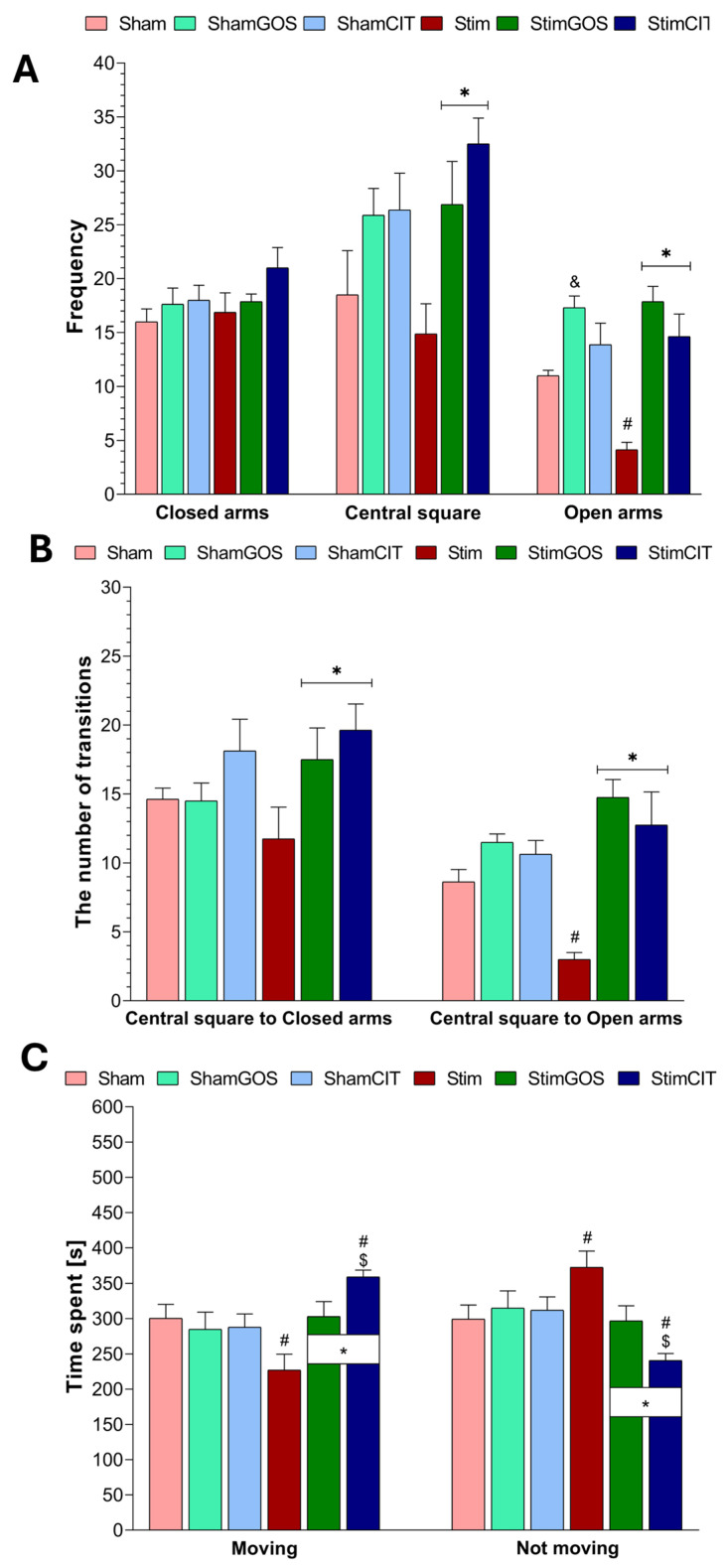
Effects of GOS supplementation or CIT administration on the anxiety level, expressed as the frequency in closed arms, the central square and open arms of the maze (**A**); the number of transitions between the central square and closed/open arms (**B**); and the time spent on moving and not moving (**C**) in the elevated plus maze in rats subjected to long-term electrical stimulation of the CeA or sham stimulation. Data are expressed as the mean ± SEM; n = 8 animals/group. Explanations: # *p* < 0.05 stimulated vs. sham rats within the same supplementation group (Stim vs. Sham; StimGOS vs. ShamGOS; StimCIT vs. ShamCIT); * *p* < 0.05 certain stimulated and supplemented groups vs. Stim; $ *p* < 0.05 StimGOS vs. StimCIT; & p < 0.05 certain sham-stimulated and supplemented groups vs. the Sham group using two-way ANOVA followed by Tukey’s post hoc test.

The high level of anxiety-like behaviour in rats was reflected by a low frequency in the open arms of the maze, a small number of the transitions from the central square to the open arms and low animal mobility (moving and not moving time duration) during the elevated plus maze test (Figure 2). Two-way ANOVA revealed that the supplementation (GOS_CIT_water) factor significantly influenced almost all tested parameters, namely the frequency in the central square and open arms of the maze, the number of transitions between the central square and closed/open arms and the time spent on moving and not moving, except the frequency in closed arms. The effect of the stimulation factor on the tested parameters was not statistically significant, but the interaction between both factors was significant for the frequency in open arms of the maze, the number of transitions between the central square and open arms and the time spent on moving and not moving (Table 2).

A low frequency in the central square and open arms of the maze, accompanied by a high frequency in the closed arms of the testing apparatus indicated a high level of anxiety. Rats subjected to long-term electrical stimulation of the CeA and without supplementation (Stim) were characterised by the lowest frequency in both the central square and the open arms of the test apparatus (Figure 2A). The frequency in the open arms of the maze in the Stim group was statistically significantly lower compared to rats subjected to sham stimulation (Sham) (*p* = 0.016). In Stim rats compared to the Sham group, the frequency in the central square was lower, and in the closed arms, it was slightly higher, although both these differences did not reach the level of statistical significance. GOS supplementation significantly increased the frequency in the central square (*p* = 0.045) and open arms (*p* < 0.001) of the maze in rats subjected to 14 days of electrical stimulation of the CeA (StimGOS) compared to Stim rats.

Citalopram administration to stimulated rats resulted in similar effects to GOS supplementation. The StimCIT group was characterised by a statistically significant higher frequency in the central square (the highest score among the other experimental groups) (*p* = 0.005) and open arms (*p* < 0.001) of the maze compared to the stimulated rats receiving only water (Stim). It is worth emphasising that unstimulated and supplemented with GOS (Sham GOS) rodents visited the open arms of the maze significantly more often than animals from the Sham control group (*p* = 0.043).

The reduced level of exploration of the new environment expressed by the small number of transitions between the central square and the open/closed arms may also reflect the increased level of anxiety in rats. Electrical stimulation of the CeA induced such an effect in Stim animals as this group showed a statistically significant smaller number of transitions from the central square to the open arms of the maze compared to Sham rats (*p* = 0.037) (Figure 2B). GOS supplementation as well as CIT administration led to the attenuation of changes induced by electrical stimulation of the CeA; stimulated and supplemented animals (StimGOS and StimCIT) were characterised by a significantly higher number of transitions from the central square to the closed arms (StimGOS: *p* = 0.039 and StimCIT: *p* = 0.006) and to the open arms (both groups *p* < 0.001) compared to the Stim group. The number of transitions between the central square and closed or open arms was similar in StimGOS compared to ShamGOS and in StimCIT compared to ShamCIT animals.

Increased anxiety levels in rats were also associated with prolonged time in which the animal did not move and reduced time spent on actively moving by the animal during the elevated plus maze test. This pattern of behaviour was observed in animals subjected to electrical stimulation of the CeA. The Stim group was characterised by statistically significant shorter time spent on moving and longer time spent on not moving (*p* = 0.013 for both parameters) compared to Sham rats (Figure 2C). GOS supplementation attenuated changes induced by electrical stimulation of the CeA; StimGOS rats spent significantly more time on moving and significantly less time on not moving (*p* = 0.010 for both parameters) compared to Stim rats. No statistically significant differences were found for these parameters between the StimGOS and ShamGOS groups. CIT administration also contributed to the alleviation of the effect of electrical stimulation of the CeA; StimCIT animals were characterised by the longest time spent on moving and the shortest time spent on not moving among all the other groups, and these results were statistically significantly different in comparison to rodents subjected to stimulation (Stim, *p* < 0.001 and StimGOS, *p* = 0.047) and in comparison to the ShamCIT group (*p* = 0.016).

Additionally, to verify the anxiolytic efficacy of GOS and CIT doses in our study, we analysed possible correlations between the frequency in the open arms in the EPM test and the mean GOS or CIT solution intake. We found that such a correlation was significant in the StimGOS and StimCIT groups (Table 3).

### 2.3. Haematological Parameters

The following parameters of the white blood cell system were analysed: leukocyte count (WBC), count and percentage of lymphocytes (LYMs), monocytes (MONs) and granulocytes (GRAs). The red blood cell system assessment included: red blood count (RBC), haemoglobin concentration (HGB) and haematocrit (HCT), mean corpuscular volume (MCV), mean corpuscular haemoglobin (MCH) and mean corpuscular haemoglobin concentration (MCHC). The analysed parameters related to platelets included platelet count (PLT), mean platelet volume (MPV), plateletcrit (PCT).

Two-way ANOVA revealed that both factors (stimulation and supplementation) significantly influenced WBC, LYM and RBC counts; HGB; HCT; and PCT. MON, GRA and PLT counts were significantly influenced only by the supplementation factor, whereas MCV and MPV were influenced only by the stimulation factor. The interaction between both factors was significant for the WBC, LYM, MON, GRA and RBC counts; HGB; HCT; PCT; and MCV. In the case of the percentage of LYM, MON and GRA, as well as MCH, MCHC and MPV, no significant effect of any of the tested factors was found (Table 4).

**Figure 3:**
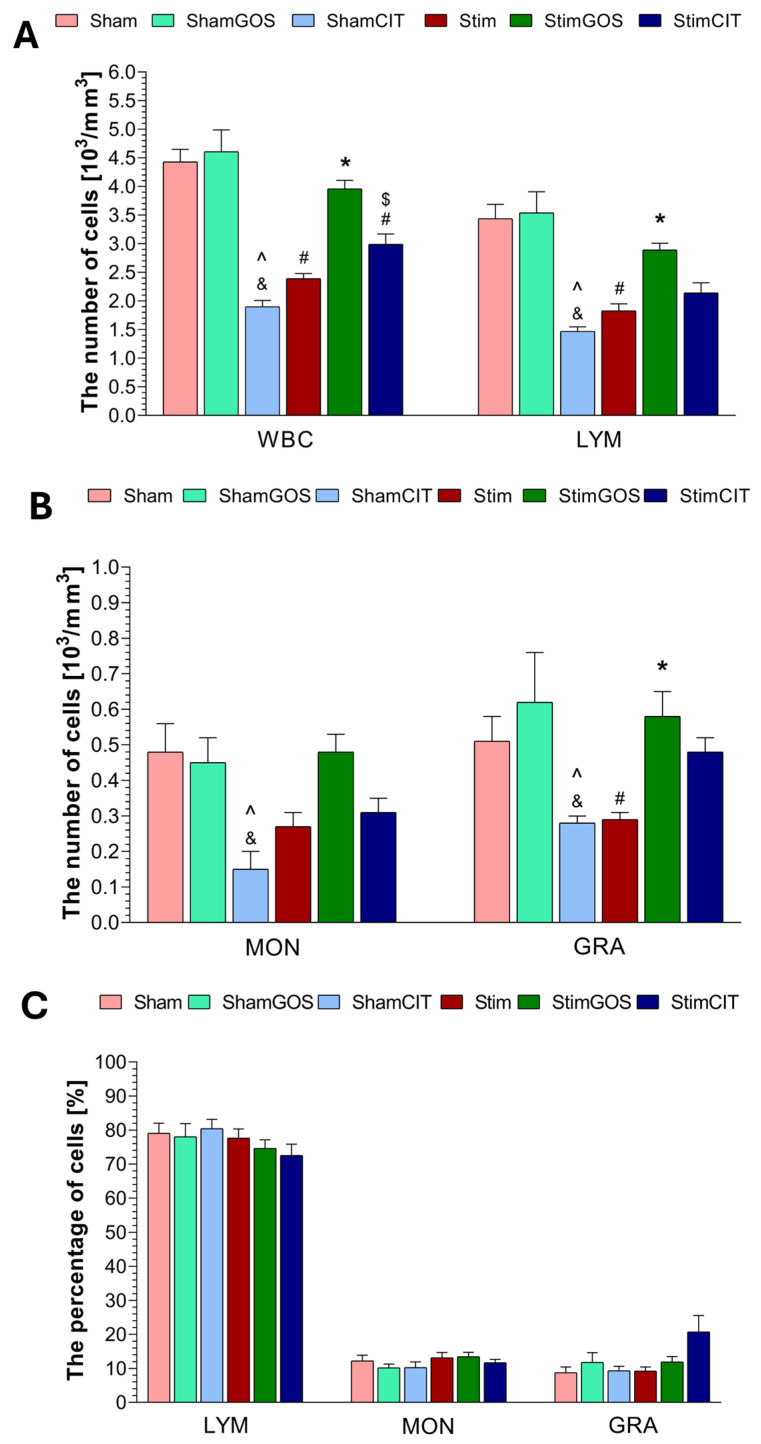
Effects of GOS supplementation or CIT administration on the white blood system: the number of white blood cells (WBCs), lymphocytes (LYMs) (**A**), monocytes (MONs) and granulocytes (GRAs) (**B**) and the percentage of LYMs, MONs and GRAs (**C**) in the peripheral blood of rats subjected to long-term electrical stimulation of the CeA or sham stimulation. Data are expressed as the mean ± SEM; n = 8 animals/group. Explanations: # *p* < 0.05 stimulated vs. sham rats within the same supplementation group (Stim vs. Sham; StimGOS vs. ShamGOS; StimCIT vs. ShamCIT); * *p* < 0.05 certain stimulated and supplemented groups vs. Stim; $ *p* < 0.05 StimGOS vs. StimCIT; & p < 0.05 certain sham-stimulated and supplemented groups vs. the Sham group; ^ *p* < 0.05 ShamGOS vs. ShamCIT using two-way ANOVA followed by Tukey’s post hoc test.

Rats subjected to 14 days of electrical stimulation of the CeA and receiving only water (Stim) were characterised by a statistically significant lower number of white blood cells (WBCs) in peripheral blood compared to Sham rats (*p* < 0.001) (Figure 3A). Due to the analysed subpopulations of WBCs, such a relationship was noted in the case of the number of lymphocytes (LYMs) (*p* < 0.001) and granulocytes (GRAs) (*p* = 0.033), while the number of monocytes (MONs) was also lower in the peripheral blood of Stim rats compared to Sham animals, but this difference was not significant (Figure 3A,B). GOS supplementation contributed to a statistically significant increase in the number of WBCs (*p* < 0.001), including LYMs (*p* = 0.012) and GRAs (*p* = 0.006), in the peripheral blood of rats stimulated and receiving prebiotics (StimGOS) compared to Stim rats. In the case of MONs, the observed difference between the StimGOS and Stim groups was not statistically significant. Interestingly, GOS supplementation led to the complete normalisation of the number of WBCs, including LYMs, MONs and GRAs, in the blood of stimulated rats (StimGOS) due to the lack of significant differences compared to rats subjected to sham stimulation and supplementation with GOS (ShamGOS). Citalopram administration to stimulated rats (StimCIT) caused a slight increase in the number of WBCs, including LYMs, MONs and GRAs, compared to the Stim group, but the observed differences were not statistically significant. StimCIT animals showed lower WBC counts, also within the analysed subpopulations, compared to the StimGOS group, and Tukey’s post hoc test revealed that only the difference in the total WBC count was statistically significant (*p* = 0.026). Sham-stimulated rats administered citalopram (ShamCIT) were characterised by the lowest number of WBCs, including LYMs, MONs and GRAs, in peripheral blood among all experimental groups. And these results were statistically significantly lower compared to Sham rats (WBC, LYM: *p* < 0.001, MON: *p* = 0.004 and GRA: *p* = 0.027) and the ShamGOS group (WBC, LYM: *p* < 0.001, MON: *p* = 0.009 and GRA: *p* = 0.002).

Intergroup differences in the percentage of LYMs, MONs, and GRAs did not reach statistical significance (Figure 3C).

**Figure 4:**
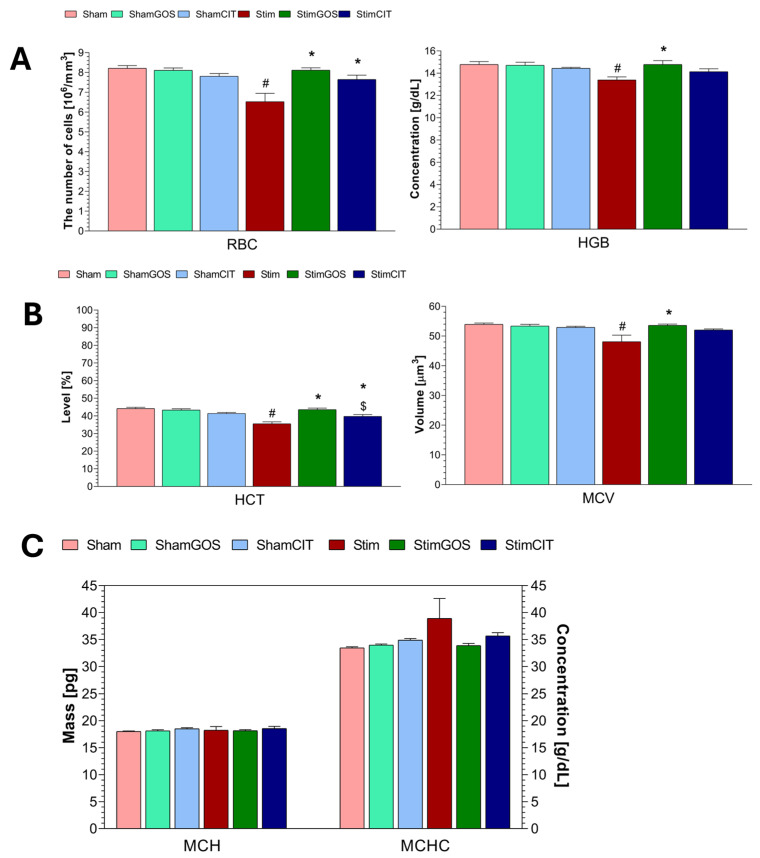
Effects of GOS supplementation or CIT administration on the red blood system: the number of red blood cells (RBCs), haemoglobin concentration (HGB) (**A**), haematocrit (HCT), mean corpuscular volume (MCV) (**B**), mean corpuscular haemoglobin (MCH) and mean corpuscular haemoglobin concentration (MCHC) (**C**) in peripheral blood of rats subjected to long-term electrical stimulation of the CeA or sham stimulation. Data are expressed as the mean ± SEM; n = 8 animals/group. Explanations: # *p* < 0.05 stimulated vs. sham rats within the same supplementation group (Stim vs. Sham; StimGOS vs. ShamGOS; StimCIT vs. ShamCIT); * *p* < 0.05 certain stimulated and supplemented groups vs. Stim; $ *p* < 0.05 StimGOS vs. StimCIT using two-way ANOVA followed by Tukey’s post hoc test.

Rats subjected to long-term electrical stimulation of the CeA and receiving water (Stim) showed the lowest number of red blood cells (RBCs) and haemoglobin concentration (HGB) in peripheral blood among all experimental groups, which also were statistically significantly lower compared to animals subjected to sham stimulation (Sham) (RBC: *p* < 0.001 and HGB: *p* = 0.008) (Figure 4A). The stim group was characterised by the lowest haematocrit (HCT) and mean corpuscular volume (MCV), and the value of these parameters was statistically significantly lower compared to Sham animals (HCT: *p* < 0.001 and MCV: *p* = 0.002) (Figure 4B). Prebiotic supplementation led to a statistically significant increase in RBC count and HGB (Figure 4A), as well as HCT and MCV (Figure 4B) in rats subjected to electrical stimulation of the CeA (StimGOS) compared to the Stim group (RBC: *p* < 0.001, HGB: *p* = 0.008, HCT: *p* < 0.001 and MCV: *p* = 0.004). The RBCs, HGB, HCT and MCV in StimGOS rats were similar to the ShamGOS group, indicating the complete normalisation of these parameters in animals stimulated by GOS supplementation.

Citalopram administration to stimulated rats resulted in a statistically significant increase in the RBC count compared to Stim animals (*p* = 0.010), while the increase in HGB was not statistically significant (Figure 4A). There were no statistically significant differences in those parameters between the StimCIT and ShamCIT groups. StimCIT rats showed a statistically significant increase in HCT (*p* = 0.011), but not in MCV compared to the Stim group (Figure 4B). In addition, the StimCIT group was characterised by a statistically significant lower HCT compared to the StimGOS group (*p* = 0.030).

Intergroup differences in the mean corpuscular haemoglobin (MCH) and mean corpuscular haemoglobin concentration (MCHC) did not reach statistical significance (Figure 4C).

**Figure 5:**
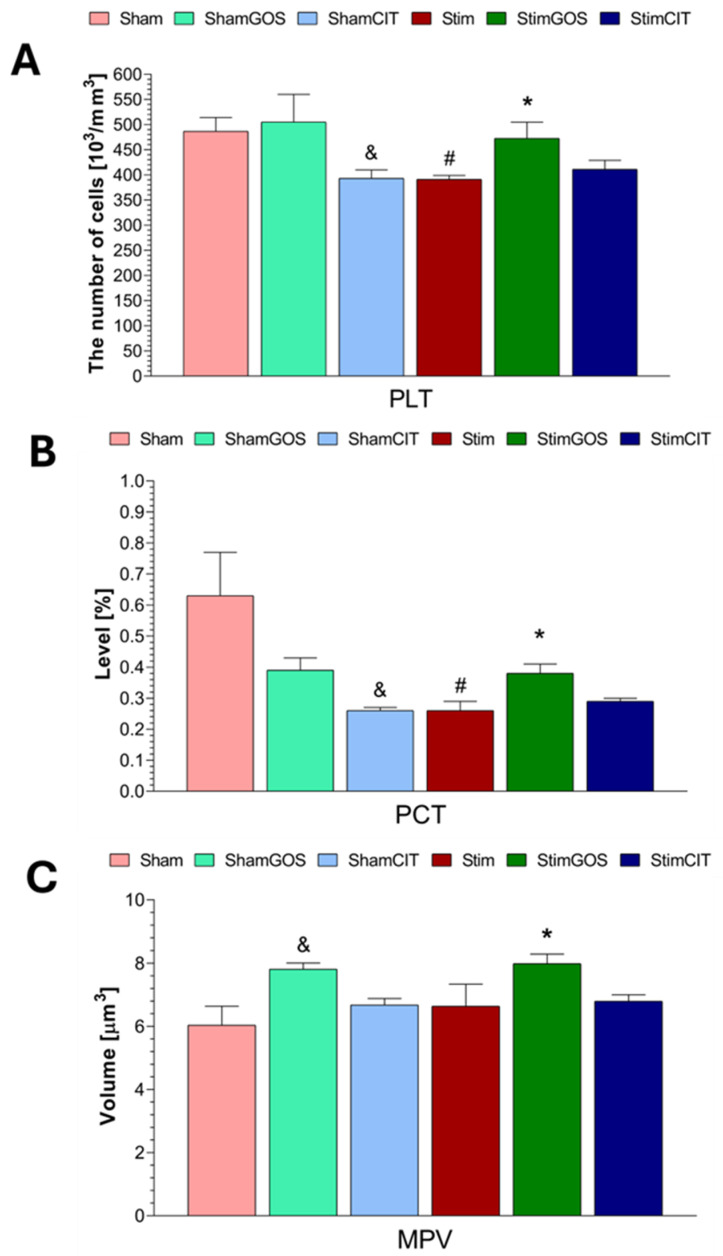
Effects of GOS supplementation or CIT administration on the platelet system: the number of platelets (PLTs) (**A**), plateletcrit (**B**) and mean platelet volume (MPV) (**C**) in the peripheral blood of rats subjected to long-term electrical stimulation of the CeA or sham stimulation. Data are expressed as the mean ± SEM; n = 8 animals/group. Explanations: # *p* < 0.05 stimulated vs. sham rats within the same supplementation group (Stim vs. Sham; StimGOS vs. ShamGOS; StimCIT vs. ShamCIT); * *p* < 0.05 certain stimulated and supplemented groups vs. Stim; & p < 0.05 certain sham-stimulated and supplemented groups vs. the Sham group using two-way ANOVA followed by Tukey’s post hoc test.

Stim rats were characterised by a statistically significant lower number of platelets (PLTs) and lower plateletcrit (PCT) in peripheral blood compared to Sham rats (PLT: *p* = 0.030 and PCT: *p* = 0.003) (Figure 5A,B). GOS supplementation led to a statistically significant increase in PLT, PCT and MPV (Figure 5C) in the StimGOS group compared to Stim animals (PLT: *p* = 0.047, PCT: *p* = 0.049 and MPV: *p* = 0.049). Additionally, GOS supplementation contributed to the complete normalisation of the platelet count, PCT and MPV in stimulated rats as the differences between the StimGOS and ShamGOS groups were not statistically significant. Citalopram administration to stimulated rats did not lead to significant changes in PLT, PCT and MPV in peripheral blood. (The StimCIT and Stim groups were characterised by similar values in these parameters.) There were no statistically significant differences between stimulated and sham-stimulated rats receiving CIT (StimCIT vs. ShamCIT) in PLT, PCT and MPV. Additionally, ShamCIT animals showed the lowest platelet counts and plateletcrit, and the differences between the ShamCIT and Sham groups were statistically significant (ShamCIT vs. Sham: PLT: *p* = 0.034 and PCT: *p* = 0.003; ShamCIT vs. ShamGOS: PLT: *p* = 0.012 and PCT: *p* = 0.043). In the case of the MPV, it was statistically significantly higher in ShamGOS rats compared to the Sham group (*p* = 0.012).

### 2.4. Plasma Immunological Markers

The following plasma immunological markers were analysed: pro-inflammatory tumour necrosis factor alpha (TNF-α) and anti-inflammatory interleukin 10 (IL-10). Two-way ANOVA revealed that the stimulation (stimulated_sham) factor and the supplementation (GOS_CIT_water) factor significantly influenced both cytokines’ plasma concentrations in rats. The interaction between both factors was also significant for TNF-α and IL-10 (Table 5).

**Figure 6:**
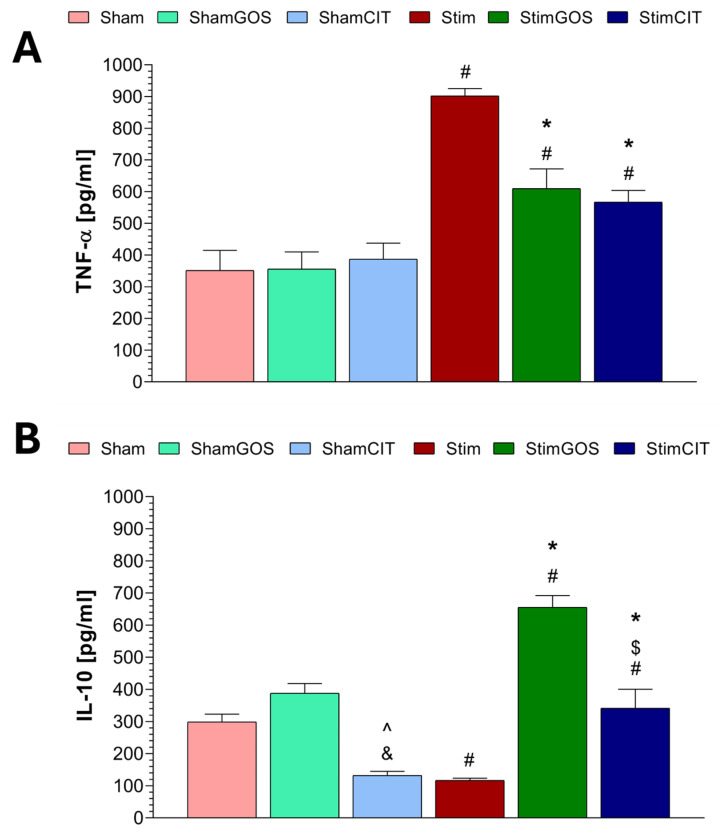
Effects of GOS supplementation or CIT administration on tumour necrosis factor alpha (TNF-α) (**A**) and interleukin 10 (IL-10). (**B**) Concentrations in the plasma of rats subjected to long-term electrical stimulation of the CeA or sham stimulation. Data are expressed as the mean ± SEM; n = 8 animals/group. Explanations: # *p* < 0.05 stimulated vs. sham rats within the same supplementation group (Stim vs. Sham; StimGOS vs. ShamGOS; StimCIT vs. ShamCIT); * *p* < 0.05 certain stimulated and supplemented groups vs. Stim; $ *p* < 0.05 StimGOS vs. StimCIT; & *p* < 0.05 certain sham-stimulated and supplemented groups vs. the Sham group; ^ *p* < 0.05 ShamGOS vs. ShamCIT using two-way ANOVA followed by Tukey’s post hoc test.

Rats subjected to electrical stimulation of the CeA and receiving only water (Stim) showed statistically significant higher concentrations of TNF-α in plasma compared to Sham animals (*p* < 0.001) (Figure 6A). GOS supplementation led to a statistically significant reduction in plasma TNF-α in rats stimulated and receiving prebiotics (StimGOS) compared to Stim animals (*p* = 0.002). However, in the StimGOS group, the concentration of TNF-α was significantly higher compared to supplemented and sham-stimulated rats (ShamGOS), which may indicate a lack of complete normalisation of the level of this pro-inflammatory cytokine as a result of prebiotic supplementation in stimulated rats (*p* = 0.011). The plasma concentration of TNF-α in animals stimulated and receiving citalopram (StimCIT) was significantly lower compared to the Stim group (*p* < 0.001) and statistically significantly higher compared to ShamCIT (*p* = 0.045). The concentration of TNF-α in the plasma of animals subjected to sham stimulation was similar, regardless of the type of supplementation (ShamGOS, ShamCIT) or its absence (Sham), and the observed differences did not reach the level of statistical significance.

Rats subjected to 14 days of electrical stimulation of the CeA, without supplementation (Stim), showed statistically significant lower plasma IL-10 concentrations compared to the Sham group (*p* = 0.009) (Figure 6B). GOS supplementation led to a statistically significant increase in plasma IL-10 concentrations in stimulated rodents (StimGOS) compared to the Stim group (*p* < 0.001). Additionally, the IL-10 level in the StimGOS group was statistically significantly higher compared to sham-stimulated and GOS-supplemented rats (ShamGOS) (*p* < 0.001). Citalopram administration to stimulated rats (StimCIT) contributed to a statistically significant increase in plasma IL-10 concentration compared to Stim animals (*p* = 0.002). The level of plasma IL-10 in the StimCIT group was also significantly higher than that of ShamCIT (*p* = 0.011) but lower than that of StimGOS (*p* < 0.001). The concentration of plasma IL-10 in animals subjected to sham stimulation and supplemented with GOS (ShamGOS) was slightly higher compared to Sham rats, and the observed difference did not reach the level of statistical significance. In the case of rodents subjected to sham stimulation and receiving citalopram (ShamCIT), the IL-10 plasma concentration was statistically significantly lower compared to the Sham (*p* = 0.032) and ShamGOS groups (*p* < 0.001).

### 2.5. Changes in Gut Microbiota

The number and diversity of gut bacteria were assessed in samples of the large intestine content collected immediately after the rats’ euthanasia. Standard microbiological methods were used (cultures on BSM and blood media and the determination of the number of bacterial colonies), as well as the MALDI-TOF method (the identification of bacterial species based on the presence of ribosomal proteins based on mass spectrometry and a comparison of the obtained results with the database). The most abundant species in the rats’ gut microbiota were *Enterococcus gallinarum*, *Enterococcus faecalis*, *Bifidobacterium animalis* and *Enterococcus casseliflavus* on the BSM medium and *Limosilactobacillus reuteri*, *Ligilactobacillus murinus* and *Lactobacillus johnsonii* on the blood medium.

The number of particular bacterial species was calculated for the number of colony-forming units in 1 mL of the sample [CFU/mL] based on the morphology of the colonies of a given species. The results are presented as a percentage of the total CFU of all identified species grown on the BSM or blood medium. Two-way ANOVA revealed that both tested factors (stimulation and supplementation) significantly influenced the abundance of *B. animalis*, *E. casseliflavus*, *L. murinus* and *L. reuteri*, and only the supplementation factor had a significant influence on the number of *E. faecalis*. In the case of the numbers of all bacterial species mentioned above, the interaction between both factors (stimulation x supplementation) was also significant. However, none of the tested factors showed a significant effect on the number of *E. gallinarum*, *E. faecalis* and *L. johnsonii*, and the interaction between both factors was significant (Table 6).

**Figure 7:**
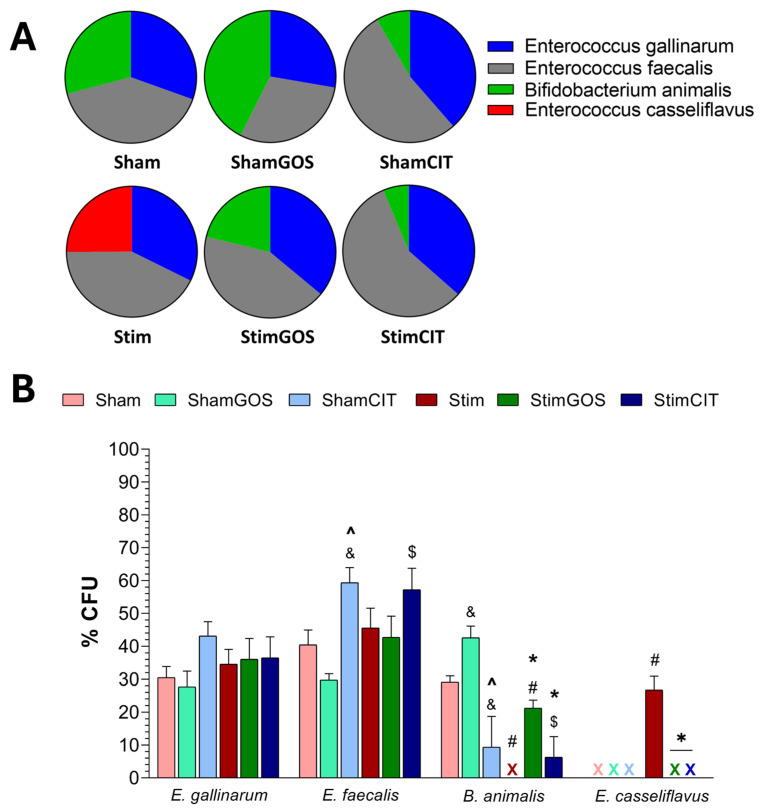
The most abundant bacterial species grown on the BSM medium (% of the total CFU of all identified species) in the gut microbiota of rats from individual experimental groups (**A**). Effects of GOS supplementation or CIT administration on the percentage (% of the total CFU) of the most abundant bacterial species (grown on the BSM medium) in the gut microbiota of rats subjected to long-term electrical stimulation of the CeA or sham stimulation (**B**). Data are expressed as the mean ± SEM; n = 6 animals/group. Explanations: X—abundance beyond the detection level (<0.1%); # *p* < 0.05 stimulated vs. sham rats within the same supplementation group (Stim vs. Sham; StimGOS vs. ShamGOS; StimCIT vs. ShamCIT); * *p* < 0.05 certain stimulated and supplemented groups vs. Stim; $ *p* < 0.05 StimGOS vs. StimCIT; & *p* < 0.05 certain sham-stimulated and supplemented groups vs. the Sham group; ^ *p* < 0.05 ShamGOS vs. ShamCIT using two-way ANOVA followed by Tukey’s post hoc test.

Microbiological quantitative and qualitative analyses of the samples of the rat large intestine content using the BSM medium showed that the gut microbiota of rats from all experimental groups contained numerous representatives of two bacterial species: *E. gallinarum* and *E. faecalis* (Figure 7A). The highest abundance of *E. gallinarum* was demonstrated in sham-stimulated rats receiving citalopram (ShamCIT) and the lowest in sham-stimulated rodents supplemented with GOS (ShamGOS); however, the intergroup differences did not reach the level of statistical significance (Figure 7B). In the case of *E. faecalis*, the highest number of representatives were found in the ShamCIT and StimCIT rats’ gut microbiota. Statistically significant fewer bacteria of this species were detected in ShamGOS compared to the ShamCIT group (*p* = 0.003), as well as in animals stimulated and supplemented with GOS (StimGOS) compared to StimCIT rodents (*p* = 0.014). Fewer representatives of *E. faecalis* were also found in the Stim group compared to the StimCIT group, but this difference was not statistically significant. *B. animalis* was abundantly represented in the microbiota of rats from the ShamGOS, Sham and StimGOS groups, and its presence was also found in animals with citalopram therapy, both the ShamCIT and StimCIT groups. This bacteria species was statistically significantly more numerous in the gut microbiota of ShamGOS animals compared to the Sham (*p* = 0.043) and ShamCIT (*p* < 0.001) groups, as well as compared to the StimGOS group (*p* = 0.007) (Figure 7B). In addition, the proportion of *B. animalis* in the large intestine of rats stimulated and supplemented with GOS (StimGOS) was also significantly higher compared to the Stim (*p* = 0.007) and StimCIT (*p* = 0.048) groups. Interestingly, in the gut microbiota of rats subjected to long-term electrical stimulation of the CeA and receiving only water (Stim), representatives of *B. animalis* were beyond the detection level (less than 0.1%), and only in this group of animals was the presence of *Enterococcus casseliflavus* found, which constituted over 25% of bacteria grown on the BSM medium (*p* < 0.001 compared to the Sham, StimGOS and StimCIT groups) (Figure 7A).

**Figure 8:**
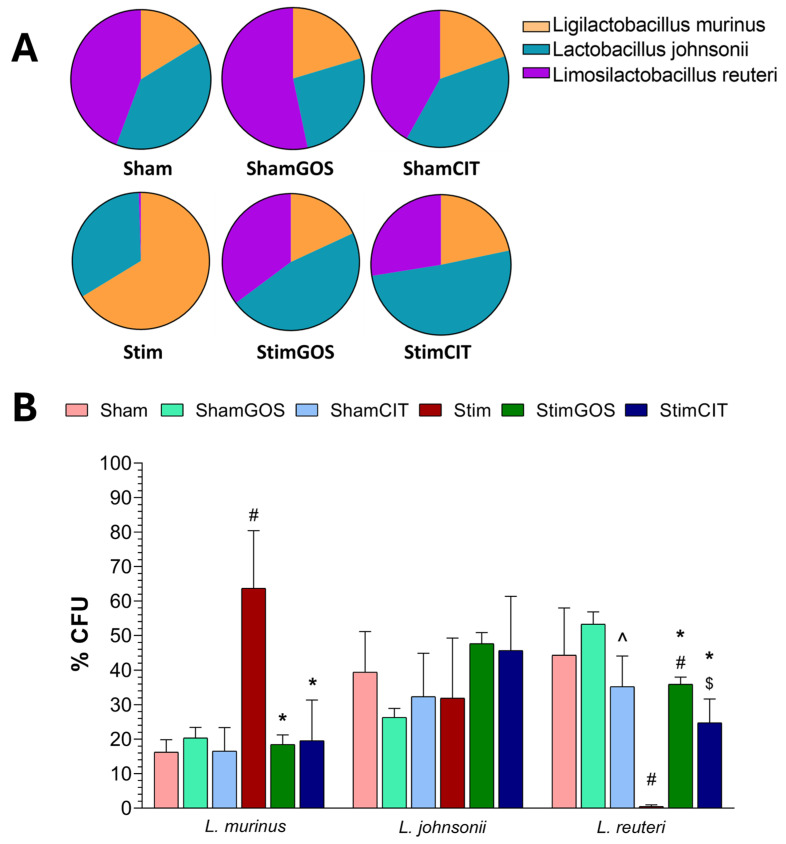
The most abundant bacterial species grown on the blood medium (% of the total CFU of all identified species) in the gut microbiota of rats from individual experimental groups (**A**). Effects of GOS supplementation or CIT administration on the percentage (% of the total CFU) of the most abundant bacterial species (grown on the blood medium) in the gut microbiota of rats subjected to long-term electrical stimulation of the CeA or sham stimulation (**B**). Data are expressed as the mean ± SEM; n = 6 animals/group. Explanations: # *p* < 0.05 stimulated vs. sham rats within the same supplementation group (Stim vs. Sham; StimGOS vs. ShamGOS; StimCIT vs. ShamCIT); * *p* < 0.05 certain stimulated and supplemented groups vs. Stim; $ *p* < 0.05 StimGOS vs. StimCIT; ^ *p* < 0.05 ShamGOS vs. ShamCIT using two-way ANOVA followed by Tukey’s post hoc test.

Microbiological quantitative and qualitative analyses of the samples of the large intestine content of rats using a blood medium showed that the dominant species were *L. reuteri*, *L. murinus* and *L. johnsonii* (Figure 8A). In the case of *L. johnsonii*, it was most abundant in the gut microbiota of animals stimulated and supplemented with GOS (StimGOS), while the lowest numbers of representatives of this species were detected in rats subjected to sham stimulation and receiving GOS (ShamGOS). However, all observed intergroup differences in the number of *L. johnsonii* did not reach the level of statistical significance (Figure 8B). *L. murinus* was the most abundant species in the gut microbiota of rats subjected to long-term electrical stimulation of the CeA and receiving only water (Stim), and this result was statistically significantly higher compared to the Sham group (*p* = 0.002), as well as to the StimGOS (*p* = 0.002) and StimCIT (*p* = 0.003) groups. The proportion of *L. reuteri* was the lowest in the gut microbiota of Stim animals, and the highest in ShamGOS rats (Figure 8A). The number of *L. reuteri* was significantly lower in the Stim group compared to Sham animals (*p* < 0.001), as well as in relation to the other groups of stimulated animals—StimGOS (*p* = 0.002) and StimCIT (*p* = 0.003). Additionally, significantly fewer representatives of this species occurred in the StimCIT rats’ microbiota compared to StimGOS (*p* = 0.041) and in ShamCIT animals compared to the ShamGOS group (*p* = 0.039) (Figure 8B).

### 2.6. Histological Verification of the Electrode Implantation

**Figure 9:**
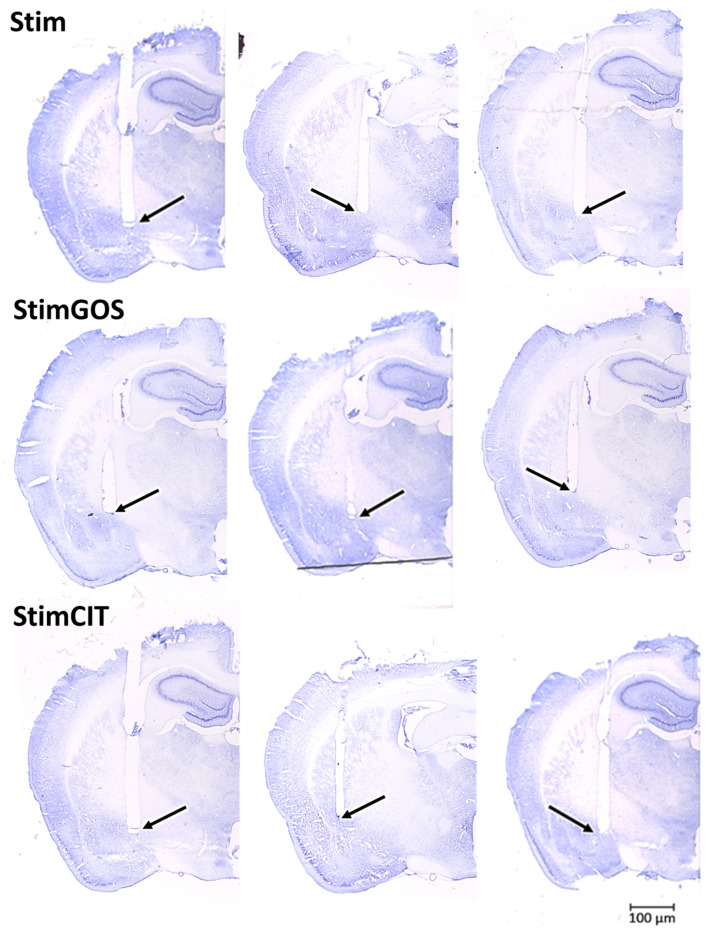
Photographs of microscopic slides showing Nissl-stained sections of the brain of representative stimulated rats. The arrow indicates the end of the stimulating electrode.

In all stimulated rats, electrical stimulation of the CeA was performed only for one hemisphere. The hemisphere was selected during screening stimulation, whereby behavioural effects were observed, such as increased motor activity (desire to escape from the actometer) or freezing. The selection of the stimulated hemisphere based on the behaviour observation was consistent with the accuracy of electrode implantation, which was confirmed through histological verification. To verify the location of the stimulating electrodes, the sections were stained using the Nissl method and then imaged using a microscope coupled with a camera (Stemi 508 + Axiocam 105 colour, Zeiss), with reference to a rat brain atlas [39]; the locations reached by the ends of the implanted electrodes were determined (Figure 9).

## 3. Discussion

In our study, the amygdala hyperactivity induced by 14 days of electrical stimulation of the central nucleus (CeA) contributed to numerous adverse changes at the behavioural, haematological and immune levels, as well as caused quantitative and qualitative disturbances in the rats’ gut microbiome. The observed changes may resemble symptoms associated with anxiety and inflammation in patients suffering from neuropsychiatric disorders such as depression, anxiety disorders or PTSD. As we have shown, GOS supplementation contributed to the normalisation of the haematological and immunological parameters and led to behavioural symptom improvements in rats subjected to CeA stimulation and supplementation (StimGOS group). In addition, the intake of this prebiotic had a positive effect on the qualitative and quantitative changes in the gut microbiota composition of stimulated rats, which may indicate a significant role of microbiota–gut–brain interactions in alleviating the effects of chronic Amg hyperactivity.

### 3.1. The Impact of Amygdala Hyperactivation and Prebiotic Supplementation on Behavioural Performance—Locomotor Activity and Anxiety-like Behaviour

The association between increased Amg activity and the induction of anxiety-like behaviour has been demonstrated in numerous studies, both using animal models and in humans [9,13,40,41]. Our results also confirm the effect of long-term electrical stimulation of the CeA on the increase in anxiety-like behaviour in rats assessed in the EPM test. Recent reports suggest that, besides the basolateral amygdala (BLA), the central amygdala, including the CeA, may be one of the key structures involved in the regulation of fear- and anxiety-related behaviour. The CeA integrates interoceptive signals from the insula and brainstem nuclei and exteroceptive signals related to stimuli initiating a fear response. Then, in close cooperation with the bed nucleus of stria terminalis (BNST), it induces an appropriate behavioural response to prevent unpleasant or threatening experiences [42]. Studies using optogenetic stimulation of BNST neurons have shown that an enhancement of anxiety-like behaviour in rats occurs via increased activity in the neuronal circuits connecting the BNST with the CeA and not other structures such as the lateral hypothalamus or the ventral tegmental area of the midbrain [43]. Asok et al. [44], using the same method to inhibit CRH neurons in the CeA acting on the BNST, confirmed that this manipulation caused disruptions in the memorization of anxiety- and fear-related stimuli. Paliwal et al. [45] confirmed that injections of GABA_A_ receptor agonists, such as muscimol, diazepam and allopregnanolone, directly into the rat’s CeA led to more frequent attendance in the open arms in the EPM test. Moreover, the injection of bicuculline (GABA_A_ receptor antagonist) into the CeA before the administration of the above-mentioned agents blocked their therapeutic effect, which suggests an important role of the CeA GABAergic neurons in the development of responses to anxiety stimuli.

An increase in anxious behaviour is accompanied by no changes in mobility or its decrease (e.g., [46,47,48]). However, it should be emphasised that in most studies using laboratory rodents, parameters related to locomotor activity (distance moved and number of crossings) are often determined during tests assessing the level of anxiety, especially in the EPM test or the open field (OF) test. Specific properties of these tests, like a large open space or bright lighting, are additionally anxiogenic for small animals [49]. In our study, we showed that rats subjected to 14 days of electrical stimulation of the CeA (Stim group) were characterised by a smaller number of transitions from the central square to both closed and open arms, as well as shorter time spent on moving in the EPM test compared to Sham animals, which is consistent with the above-mentioned literature reports. The actual assessment of the influence of CeA electrical stimulation on the locomotor activity in our study was based on the number of horizontal, vertical and ambulatory movements counted in the actometers (a non-anxiogenic environment with standard light conditions). The obtained results indicate that long-term electrical stimulation of the CeA contributed to an increase in the rats’ locomotor activity to the basal level of mobility (before stimulation). Additionally, just after 7 days of stimulation, the motor activity of the stimulated rats (Stim group) was significantly higher compared to the sham-stimulated rats (Sham group), and after another week of this procedure, it increased even more. In contrast, in the control groups (Sham, ShamGOS and ShamCIT), the mobility of the animals gradually decreased, which may be related to adaptation to the procedure and environment conditions [50]. It is worth emphasising that the CeA sends monosynaptic inhibitory projections to the ventrolateral part of the periaqueductal gray (PAG), which mediates signal transmission to the midbrain and spinal cord motor areas to induce appropriate locomotor responses [51]. The involvement of the superior colliculus parvalbumin neurons may also be important in the modulation of motor functions by the CeA. The excitatory signal from this structure is sent to the BLA via the thalamic posterior lateral nucleus and to the CeA via the parabigeminal nucleus. It has been proven that the activation of the superior colliculus–parabigeminal nucleus–CeA neuronal circuit initiates the behavioural pattern of the escape–freezing response, while the activity of the BLA is primarily associated with the freezing response, which is not accompanied by escape [51,52]. It can be assumed that the hyperactivity of Amg induced in our study via electrical stimulation of the CeA reflected contact with an unpleasant or potentially threatening stimulus and contributed to the implementation of an escape response in Stim rats with the highest horizontal and vertical locomotor activity. Similar behavioural effects were observed after long-term electrical stimulation of the BNST in our previous work [12], which may indicate an important role in the enhanced activation of the CeA-BNST connection in locomotor processes related to the escape response.

Our results indicate an anxiolytic effect of GOS. Three weeks of supplementation with these prebiotics led to the complete normalisation of the anxiety-like behaviour measured in the EPM test in rats subjected to long-term electrical stimulation of the CeA. Similar behavioural effects have been demonstrated in other preclinical studies using GOS [30,32,34] or a mixture of GOS with other prebiotics [32,53,54,55]. The anxiolytic properties of GOS have also been confirmed in several human studies [31,33,56]. The effect of GOS on anxiety-related behaviour is thought to be mediated through their ability to stimulate the growth and activity of native probiotic bacterial species from the *Bifidoacterium* and *Lactobacillus* genera. These in turn produce beneficial metabolites, including neurotransmitters modulating anxiety (GABA, serotonin and norepinephrine), enhance immune functions related to anti-inflammatory processes, and modulate the stress response [57]. Bravo et al. [58] demonstrated that probiotic supplementation with the *L. rhamnosus* JB-1 strain can contribute to reducing the level of anxiety-like behaviour in mice via the vagus nerve, leading to changes in GABA receptor mRNA expression in a brain structure- and receptor subtype-dependent manner. It should be emphasised that GABA receptors are strongly involved in the induction of proper fear- and anxiety-related responses and constitute the therapeutic target of some anxiolytic drugs [58]. Importantly, none of the above-mentioned beneficial effects of supplementation were observed in animals subjected to vagotomy.

The influence of prebiotics on locomotor activity is still poorly known, although considering that such fibres mediate beneficial neuroactive metabolite production by intestinal probiotic bacteria, it can be assumed that they modulate the activity of the HPA axis and autonomic and central nervous system regions associated with motor functions. Savignac et al. [30] evaluated the effects of prophylactic 3-week GOS supplementation on changes induced by a single intraperitoneal injection of a lipopolysaccharide (LPS) in mice. Measurements of locomotor activity were performed in the OF test 4 h after LPS administration. Although GOS administration contributed to increased locomotor activity in LPS-injected animals compared to water-treated mice, the observed difference was not statistically significant. Our results indicate that 3 weeks of GOS supplementation significantly attenuated vertical and ambulatory locomotor hyperactivity in rats induced by long-term electrical stimulation of the CeA and led to a complete normalisation of the mobility level in the horizontal plane. Similar effects were demonstrated by Osman et al. [59], confirming that 2 weeks of supplementation with a mixture of bioactive polyphenols with prebiotic properties significantly reduces the level of locomotor activity induced by intraperitoneal morphine injections. The effect of prebiotics on locomotor activity is therefore not unidirectional but is usually associated with the normalisation of locomotor activity increased or decreased by various unfavourable factors [60,61,62].

### 3.2. The Impact of Amygdala Hyperactivation and Prebiotic Supplementation on Haematological Parameters

The obtained results of the rats’ peripheral blood morphology indicate that long-term electrical stimulation of the CeA leads to disturbances of numerous haematological parameters. Changes in the red blood cell system induced by chronic hyperactivity of the Amg manifested as a decrease in the red blood cell (RBC) count, haemoglobin (HGB) concentration, haematocrit (HCT) level and mean corpuscular volume (MCV) in Stim rats compared to Sham animals, which may indicate anaemia. In the context of anxiety- and stress-related neuropsychiatric disorders, available data on RBC system abnormalities are limited. Studies in 378 hospitalised patients with neuropsychiatric disorders have shown that more than 25% of them had anaemia as a co-occurring disease [63]. This result is significantly higher than the estimated anaemia prevalence rate of 5.6% in the USA population [64]. The results of the peripheral morphology of 2381 neuropsychiatric patients suffering from schizophrenia (52%), depression (33%) and bipolar disorder (15%) hospitalised in 2011–2016 were analysed by Wysokiński i Szczepocka [65]. Patients with schizophrenia were characterised by a significantly higher RBC count and HCT compared to those with depression and bipolar disorder. Interestingly, significantly more patients with depression and bipolar disorder showed a decrease in RBC count, HGB and HCT values, which indicate anaemia. Zhang et al. [66] confirmed that patients with mild cognitive impairment and higher Hamilton scale scores indicating higher anxiety levels had a lower number of RBC and HGB concentration in peripheral blood. It is considered that the main cause of observed abnormalities may be the co-occurring processes associated with inflammation, which contribute to erythropoiesis inhibition through the influence of pro-inflammatory cytokines on erythrocyte progenitor cells or the production of erythropoietin [67].

There are several reports on the relationship between neuropsychiatric disorders and disturbances of the platelet system. We showed that rats subjected to 14 days of electrical stimulation of the CeA were characterised by significantly lower platelet (PLT) counts and plateletcrit (PCT) compared to Sham control animals. Wei et al. [68] reported similar changes and additionally observed that the mean platelet volume (MPV) was significantly higher in hospitalised patients with depressive disorders compared to healthy individuals. The abnormalities mentioned above were also demonstrated by Almis and Aksoy [69] in patients with generalised anxiety disorder (GAD) compared to the control group. Wang et al. [70] demonstrated that in patients with depression compared to healthy individuals, only MPV was significantly increased, and no significant differences in PLT and PCT were observed. As with the RBC system, inflammation may be one of the main factors influencing platelet system abnormalities. It has been suggested that as a result of the increased concentration of pro-inflammatory cytokines, the number of platelets is reduced due to the activity of immunological effector cells [71]. The inconclusiveness of these results and the fact that platelets may exhibit pro-inflammatory activity [68] indicate the need for further research on the relationship between the platelet system and neuropsychiatric disorders.

A meta-analysis of results on white blood cell (WBC) system parameters showed that the peripheral blood WBC count, including the number of granulocytes (GRAs), monocytes (MONs) and lymphocytes (LYMs), was significantly higher in patients with depression compared to healthy individuals [72]. The results obtained in our study are inconsistent with these reports. We showed that in the peripheral blood of rats subjected to long-term electrical stimulation of the CeA (Stim group), there are significantly fewer LYMs and GRAs, which is also reflected in the lower WBC count as compared to Sham animals. It should be noted that in our study, the underlying cause of haematological changes was chronic Amg hyperactivity, which may contribute to chronic stress response stimulation, leading to immunosuppression. Han et al. [73] observed that oral administration of norepinephrine (100 mg/kg bw) in rats reduced the number of WBCs and neutrophils and increased the number of LYMs, compared to animals that received saline. Kishor et al. [74] showed that a 10-day chronic behavioural stress procedure increased the WBC count in the rats’ peripheral blood, while McDonald et al. [75] noted that under similar conditions of chronic stress in mice, the number of MONs and LYMs was significantly lower. In our previous study, we demonstrated that the number and cytotoxic activity of NK (natural killer) cells increased, whereas the number of WBCs and their subpopulations did not change in rats subjected to long-term electrical stimulation of the BNST [12]. In summary, changes in the WBC system co-occurring with increased levels of anxiety and stress are ambiguous, and the mechanisms of these interactions require further research.

The modulation of gut microbiota by psychobiotics (prebiotics, probiotics or a combination thereof, known as synbiotics) has beneficial effects on host immune functions [26], and there are several literature data that indicate their positive influence on haematological parameters. A significant increase in the number of WBCs, including LYMs, was observed in the peripheral blood of healthy rats after 3 weeks of supplementation with an aqueous extract of *Vernonia amygdalina* leaves, the main prebiotic components of which are fructooligosaccharides (FOSs) and GOS [76]. Sanguri and Gupta [77] demonstrated that prophylactic administration of mannanoligosaccharides (MOSs) two hours prior to radiotherapy effectively minimises radiation-induced damage of the haematopoietic system and accelerates the regeneration of circulating cells in mice peripheral blood. The number of WBCs and LYMs in radiotherapy-treated mice receiving MOS was significantly higher compared to unsupplemented animals, and this effect was maintained for the next 30 days. Rehman et al. [78], in clinical trials involving infants suffering from severe malnutrition, showed that 48 days of GOS supplementation contributed to a significant increase in the level of haematological parameters (HGB, HCT and WBC) compared to the control group. The beneficial effect of synbiotic supplementation on improving RBC system disorders in patients suffering from anaemia and hypercholesterolemia has also been proven [79,80]. The mentioned data are consistent with our results, which confirmed that 21 days of GOS supplementation led to the complete normalisation of haematological parameter abnormalities induced by long-term electrical stimulation of the CeA in rats. It can be assumed that prebiotics, through native probiotic species inhabiting the host microbiome, regulate the activity of signalling pathways related to the protection of cells against damage, including haematopoietic stem cells [77]. Moreover, the effect of prebiotics on increasing the number of probiotic bacterial species enhances signals received by TLR receptors, the presence of which has been confirmed on various types of host cells [81,82,83]. Interestingly, TLR-mediated signalling can modulate mitochondrial activity and stimulate antioxidant processes, thus promoting cytoprotective mechanisms [84].

### 3.3. The Impact of Amygdala Hyperactivation and Prebiotic Supplementation on Plasma Immunological Markers

Various studies have shown an association between Amg hyperactivity and inflammation. Individuals with higher levels of pro-inflammatory cytokines in plasma induced by bacterial antigen administration had concomitant increased Amg neuronal activity [85]. A similar effect was induced by stress exposure, leading to increased levels of peripheral inflammatory markers and Amg hyperactivity [86]. Interestingly, adolescents who were characterised by increased Amg activity after exposition to human faces expressing fear and anxiety also showed increased levels of TNF-α [87]. These data suggest an immunomodulatory effect of this structure in response to stimuli related to stress, anxiety or immunisation [88]. Moreover, significantly increased levels of inflammation-related markers have been reported in patients with depression, anxiety disorders and PTSD, and the development and course of Amg hyperactivity play an important role [8,89]. Our results also indicate an increase in inflammatory processes and a decrease in anti-inflammatory activity in rats with induced Amg hyperactivity (Stim group). In the plasma of rats subjected to 14 days of electrical stimulation of the CeA, we demonstrated a significantly higher level of TNF-α and a reduced level of IL-10 compared to the Sham group. Ming et al. [90], using patch-clamp recordings of neuronal activity in rat brain slices containing Amg, demonstrated that TNF-α administration to the CeA reduced the threshold for triggering action potentials in neurons. The authors did not observe this effect in the presence of a glutamate receptor antagonist, which may indicate the involvement of glutamate in mediating changes induced by TNF-α. Therefore, it can be assumed that Amg is particularly sensitive to increased TNF-α levels. It has also been established that, through its effects on microglia, this cytokine stimulates corticotropin-releasing hormone neurons in the CeA.

Increasing evidence suggests that prebiotics have a beneficial impact on the regulation of inflammatory processes. It has been confirmed that the administration of the FOS+GOS in mice with induced obesity mixture leads to a reduction in the plasma levels of IL-1β, IL-6 and TNF-α, as well as other inflammatory markers, including the p65 subunit associated with the NFκB pathway, nitric oxide synthase and cyclooxygenase 2 [55,91]. Tang et al. [34] showed that GOS supplementation contributed to a reduction in the plasma levels of pro-inflammatory cytokines such as IL-4, IL-13, IFN-γ, and TNF-α in a mouse model of atopic dermatitis. Similar effects were reported in a mouse model of LPS-induced peripheral immunisation [30,92]. An intensification of the anti-inflammatory response manifested by increased plasma IL-10 concentrations following supplementation with GOS or a mixture of GOS with other prebiotics has been demonstrated in animal models of inflammatory diseases [55,93] and in human studies [94,95]. The presented data are consistent with our results. We demonstrated that GOS supplementation significantly alleviated the inflammatory processes induced by long-term electrical stimulation of the CeA, which was reflected in a reduction in plasma TNF-α concentration (StimGOS group). The GOS also increased the anti-inflammatory response expressed by higher plasma IL-10 levels in StimGOS rats compared to stimulated and unsupplemented animals (Stim group). One of the mechanisms responsible for the above-mentioned beneficial effects exerted by GOS may be the promotion of lymphocyte differentiation into a regulatory T-cell phenotype capable of IL-10 production, as observed in in vitro studies [96]. Additionally, short-chain fatty acids (SCFAs) produced by probiotic species of gut bacteria inhibit the activity of signalling pathways associated with inflammatory processes, e.g., TLR4/NFκB [97].

A GOS affects CNS functions mainly through its influence on gut microbiota composition by increasing the numbers and activity of probiotic bacteria representatives. In turn, these newly proliferated intestinal probiotics and their neuroactive metabolites (e.g., SCFAs) exhibit a variety of neurobiological mechanisms, including the alteration of neurotransmitter levels, the regulation of the HPA axis, and a reduction in neuroinflammation [18,19,28,57].

In the context of neuroinflammation and neurological disorders, SCFAs can improve the compromised blood–brain barrier by affecting the transcription of junctional complex genes, influencing their epigenetic mechanisms and finally increasing tight junction protein expression [98]. Moreover, research by Gao et al. [99] emphasises the protective role of SCFAs against astrocytic activation and related neuroinflammatory responses, especially those related to the SGK/IL-6 pathway. It can be assumed that prebiotics, including GOS, have a direct and indirect effect on inflammatory processes by silencing excessive pro-inflammatory responses and stimulating anti-inflammatory mechanisms.

### 3.4. The Impact of Amygdala Hyperactivation and Prebiotic Supplementation on Gut Microbiota Composition

Studies involving patients with neuropsychiatric disorders, as well as results of studies using germ-free (GF) and antibiotic-treated animals, emphasise the important role of gut microbiome dysbiosis in the development of symptoms associated with anxiety, depression or an abnormal response to stress [19,20,21,22]. The obtained results indicate that long-term electrical stimulation of the CeA induced qualitative and quantitative disturbances in the rat gut microbiota. The presence of *Enterococcus casseliflavus* species was noted only in Stim animals, with a simultaneous reduction in the number of *Bifidobacterium animalis* representatives (below the detection level 0.1%). Moreover, within the *Lactobacillaceae* species, the number of *L. murinus* was significantly higher, and *L. reuteri* was significantly lower compared to animals subjected to sham stimulation. *E. casseliflavus*, despite belonging to native Enterococci inhabiting the gut microbiome, shows high resistance to antibiotics [100] and may be associated with infections due to decreased immunity [101]. *L. reuteri* has bacteriostatic properties by inhibiting pathogenic species proliferation. Therefore, a reduction in the number of this species may indicate the weakening of the processes aimed at maintaining the proper microbiological balance in the intestines [102]. Additionally, it has been proven that antibiotic-induced dysbiosis can lead to excessive growth of *L. murinus*, impairing intestinal metabolic functions [103]. Furthermore, animals fed a high-fat diet also showed *L. murinus* dominance, reduced SCFA production, and increased levels of inflammatory markers [104].

In our study, we demonstrated that GOS supplementation contributed to an increase in the abundance of intestinal probiotic bacteria, including *B. animalis* and *L. reuteri*, which is consistent with the literature data [29,34,56,94]. The highest abundance of both species was found in the microbiota of rats subjected to sham stimulation and supplemented with GOS (ShamGOS group). However, in the stimulated and supplemented (StimGOS) animals, these bacteria were significantly more numerous compared to the rats subjected to electrical stimulation of the CeA and receiving only water, which may indicate an important role of GOS in protection against dysbiosis and/or in the reconstruction of the proper composition of the gut microbiota under conditions of chronic Amg hyperactivity. It can be assumed that GOS, as the basic food source for probiotic bacteria, stimulated their proliferation. As a result, these bacteria could compete more effectively for a niche and displace potentially pathogenic native and exogenous species (colonisation resistance). Moreover, GOS, by increasing the proportion of probiotic bacteria, could contribute to immune mechanism enhancement, thereby protecting the host organism against infections, including those caused by pathogenic bacteria. *L. reuteri* supplementation has been shown to increase the expression of genes associated with anti-inflammatory responses and simultaneously reduce the levels of gene expression related to pro-inflammatory responses [105]. Additionally, Mazzoli et al. [106] recently reported that this species of bacteria may exhibit neuroprotective properties that have a beneficial effect on the hippocampus of rats in which peripheral and central disorders were induced by a high-sugar and high-fat diet. Our results suggest that the presence of numerous representatives of this species in the StimGOS rats’ gut microbiota could have contributed to the increased activation of mechanisms that alleviate inflammation induced by electrical stimulation of the CeA, which may affect the observed behavioural deficit improvements.

### 3.5. Comparison of Galactooligosaccharides’ and Citalopram’s Therapeutic Potential

Many studies using animal models and involving patients with neuropsychiatric disorders have confirmed that psychobiotics are similarly effective as conventional pharmacological therapies in alleviating symptoms of these diseases [107]. The main mechanism of action of SSRIs is their impact on the serotonergic system. It has been proven that prebiotics, including GOS, and their metabolites can also influence this neurotransmission system by enhancing serotonin production through the modification of tryptophan metabolism in both the gut and the brain [30,107]. Our results also confirm the high efficacy of GOS, which is comparable to antidepressants and the anxiolytic SSRI drug citalopram, in alleviating changes induced by electrical stimulation of the CeA. Importantly, in several studied aspects, the therapeutic effects of GOS supplementation were greater than those of CIT therapy. GOS supplementation was significantly more effective in attenuating haematological abnormalities in StimGOS rats compared to StimCIT animals. It has been suggested that psychotropic drugs, including selective serotonin reuptake inhibitors (SSRIs), used in chronic neuropsychiatric diseases cause haematological side effects such as anaemia, leukopenia, thrombocytopenia or thrombocytosis. Moreover, the occurrence of these adverse effects is more common in patients with long-term therapy [63]. The impact of SSRI drugs on the gut microbiota should also be taken into account. In our study, we showed that in both groups of animals receiving CIT, the numbers of *B. animalis* and *L. reuteri* were significantly lower, and *E. faecalis* was significantly higher compared to rats supplemented with GOS (ShamCIT vs. ShamGOS and StimCIT vs. StimGOS, respectively). It has been reported that SSRIs, such as CIT, may alter the composition of the gut microbiota [108]. Ait Chait et al. [109] demonstrated that many psychotropic drugs exhibit antibacterial activity against species isolated from the gut microbiome. This evidence may be relevant for the design of clinical trials to evaluate the therapeutic efficacy of psychobiotics in patients suffering from neuropsychiatric disorders during concomitant conventional pharmacological therapy.

For the first time, this work evaluated the early effects of GOS supplementation in a rat model of amygdala hyperactivation induced by electrical stimulation. However, our study has some limitations. One is the lack of follow-up data on the lasting therapeutic effect of GOS supplementation after treatment cessation. Furthermore, this study has not tested the changes in intestinal permeability or structures. Indeed, a beneficial effect of psychobiotics, including GOS, on strengthening the integrity of the intestinal–blood barrier (increased expression of tight junction proteins, e.g., occludin and ZO-1) and improving the epithelium condition (more villi and goblet cells and increased mucin production) was reported [97,110]. Finally, the study of sex differences in the effectiveness of GOS in the context of anxiety disorders and amygdala hyperactivity could be a valuable addition to this work due to the reduced fear extinction and cholinergic activity in the amygdala of female rats as compared to males [111]. All these issues should be addressed in future research.

## 4. Materials and Methods

### 4.1. Animals and Experimental Approach

All animal procedures were carried out in accordance with the European Parliament and the Council of 22 September 2010 (2010/63/EU), as well as under the authority of the Local Ethical Committee for the Care and Use of Laboratory Animals at the University of Technology in Bydgoszcz, Poland (decision number 38/2017). According to this decision, the welfare of the animals was monitored during the experiment. The observation of the rats for atypical behaviour (e.g., stereotyped movements, a lack of rearing, etc.) and pain was conducted daily during recovery from stereotaxic implantation, the stimulation period (before stimulation and after returning to home cages) and GOS or CIT supplementation. The body weight, skin, stool consistency, and urinary signs were analysed once a week. The endpoints of the procedure (criteria of early euthanasia for humanitarian reasons) were planned if the body weight loss exceeded 10% or if the animal exhibited behaviours that impaired its ability to perform essential activities, such as free exploration, grooming or food and water intake. Throughout this experiment, no symptoms were observed that would warrant the early termination of the procedure.

Male Wistar rats that were 3 months old (n = 48) were used in our studies (Tri-City Central Animal Laboratory, Research Service Centre of the Medical University of Gdansk, Poland). For the duration of the experiment, rats were maintained under standard conditions in the Certified Conventional Vivarium of the Department of Animal and Human Physiology at University of Gdansk and housed separately in polycarbonate cages (20 cm width, 37 cm length and 18 cm height) with eye contact and olfactory communication with each other on a 12 h light/dark cycle (lights on at 6.00 a.m.), with the temperature maintained at 22 °C and the humidity at 50–60%. Water and food were available *ad libitum*.

**Figure 10:**
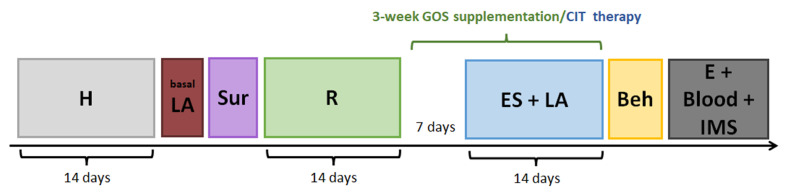
Diagram of the experimental procedures. Explanations: H—handling; basal LA—basal locomotor activity measurement using actometers; Sur—stereotaxic surgery of stimulation electrode implantation to the CeA; R—recovery; 3-week GOS supplementation/CIT therapy started 7 days before the first electrical stimulation of the CeA; ES + LA—electrical stimulation of the CeA using actometers with locomotor activity measurements; Beh—anxiety-like behaviour assessments in the elevated plus maze test after the last stimulation session; E + Blood + IMS—euthanasia, blood and intestinal microbiota sample collection.

After a two-week habituation period, all animals were subjected to further procedures according to the diagram presented in Figure 10.

### 4.2. Stereotaxic Surgery for Stimulating Electrode Implantation

After the adaptation period, all rats were subjected to stereotaxic surgery, during which stimulating electrodes were implanted bilaterally (in the left and right hemispheres) into the central nucleus of the amygdala (CeA). Stimulating electrodes were handmade in our laboratory, as described by Jerzemowska et al. [112]. Briefly, the monopolar stimulating electrode had a 0.28 mm diameter, was made from an approximately 8 mm long stainless-steel wire and was coated with epoxy varnish, except for the flat-cut end, which was inserted into the brain structure. A gold-plated male amphenol connector was soldered to the other end of the electrode. The reference electrode consisted of a jeweller’s screw wrapped with a highly conductive silver wire with a soldered male amphenol connector. The counterpart of the amphenol connector was soldered to the stimulator cable.

The method of stereotaxic implantation of electrodes into the rat brain structures is commonly used in the Department of Animal and Human Physiology [12,14,112,113,114]. Briefly, rats under isoflurane inhalation anaesthesia (3% mixture with oxygen using an isoflurane vaporizer, Rothacher-Medical, Switzerland, and oxygen pump, Bitmos OXY 6000, Bitmos GmbH, Düsseldorf, Germany) with a subcutaneous injection of an analgesic (Butomidor, Richter Pharma AG, Wels, Austria) at a dose of 0.4 mg/kg bw were mounted in a stereotaxic apparatus for small animals (Hugo Sachs Elektronik, March-Hugstetten, Germany). After exposing the skull bones, bregma and lambda points were determined. The coordinates for stimulation electrode implantation into the CeA were determined based on a rat brain atlas [39]: AP = 2.1 mm posterior to the bregma, L = 3.8 mm lateral to the midline and D = 7.8 mm ventral from the skull surface. Small holes were drilled in the skull bones using a precision trepanation drill in designated locations. Four additional drillings were made, two at the front and two at the back of the exposed surgical field, as they were necessary to insert the reference electrode (front of the skull) and anchor screws (front and back of the skull). All electrodes and anchor screws were fixed solidly to the skull of the animal using dental acrylic cement (Duracryl, SpofaDental, Warszawa, Poland). Once the acrylic had set, the scalp was gently pulled over the implant and sewn with absorbable surgical stitches (Safil, B. Braun Medical Inc., Melsungen, Germany). After surgery, each rat was subjected to an intramuscular injection of an antibiotic gentamicin in a dose of 4 mg/kg bw (Biowet. Puławy, Poland) as a prophylactic procedure to avoid infection. Next, the animal was gently placed in a cage and moved to a warm room, and when the animal awoke from anaesthesia, it was moved to a cage in the animal vivarium. The post-surgical recovery period was two weeks.

### 4.3. Pre-Stimulation Screening and Experimental Groups

At the end of the second week of recovery (12–14 days after surgery), the rats were randomly assigned to the electrical stimulation (n = 24) and the sham stimulation (n = 24) groups. Then, in the electrical stimulation group, a pre-stimulation screening was performed to establish stimulation parameters individually for each rat by observing the behaviour of the animal during current flow. Screening was performed once for each rat using a 215/I stimulator (Hugo Sachs Elektronik D7 806 March-Hugstetten, Germany) in an acoustically isolated room for behavioural studies. During this procedure, the current intensity value was selected for each hemisphere of a tested rat, at which behaviours such as locomotor arousal or freezing, flinching, piloerection, and trembling of the snout and/or paws contralateral to the stimulated structure were observed. The initial current intensity during screening was 30 μA, and in the absence of the above-mentioned behavioural responses, the intensity was increased by 5–10 μA; the procedure was performed for several stimulation cycles (30 s current on + 20 s off) while not exceeding the target value of 80 μA (the limiting intensity value at which behavioural responses were usually observed). Rectangular pulses with a single pulse width of 0.1 ms and a frequency of 50 Hz were used during stimulation.

Within the electrical stimulation and sham stimulation groups, the animals were randomly assigned to the supplemented groups (GOS or CIT) or those receiving only water. There were 6 experimental groups: (1) Sham—rats subjected to sham stimulation and receiving water (n = 8), (2) ShamGOS—rats subjected to sham stimulation of the CeA and GOS supplementation (n = 8), (3) ShamCIT—rats subjected to sham stimulation of the CeA and CIT therapy (n = 8), (4) Stim—rats subjected to electrical stimulation of the CeA and receiving water (n = 8), (5) StimGOS—rats subjected to electrical stimulation of the CeA and GOS supplementation (n = 8) and (6) StimCIT—rats subjected to electrical stimulation of the CeA and CIT therapy (n = 8).

### 4.4. GOS Supplementation and CIT Therapy

Both tested compounds were administered in the drinking water, and supplementation started a week before electrical stimulation of the CeA (the 15th day after the surgery). To estimate the amount of fluid consumption, the drinking bottles with water were weighed each day during a two-week recovery period. The rats drank about 30 g of water per day. However, each supplemented rat was given a volume of approximately 150 mL of GOS or CIT solution to freely consume fluid, which was monitored daily by weighing drinking bottles before administration and after 24 h. GOS supplementation, as well as CIT administration, lasted for 3 weeks, until the day of euthanasia.

The commercially available Bimuno^®^ powder (Clasado Biosciences, Berkshire, UK) containing a patented mixture of galactooligosaccharides (48%) with other sugars, such as lactose (26%), glucose (14%) and galactose (12%), was used. The GOS solution (13 g/L) was freshly prepared each day and poured into sterilised drinking bottles. The GOS dosage for a rat per day was approximately 1.3 g/kg bw. Savignac et al. [30] showed that such GOS concentration in drinking water and a 3-week supplementation period resulted in a significant increase in the number of probiotic *Bifidobacteria* species in the animals gut microbiota and also reduced anxiety-related behaviour induced by peripheral immunisation. In addition, it was proven that a mixture of sugars in the same proportion, in the absence of GOS, did not show similar effects in animals. A 3-week period of supplementation with Bimuno^®^ at 0.5–1.5 g/kg/day has also been confirmed to be effective in more recent studies using animals [115,116,117]. The most commonly used dose in humans, depending on the supplementation period, is 3–15 g per day (0.05–0.25 g/kg/day), and the gut transit speed in humans is lower than that of rats; therefore, higher doses of prebiotics (per kg) are often used in animal studies to compensate and allow for enough interaction time with the gut microbiota [31,56,94,118].

The commercially available selective serotonin reuptake inhibitor drug (SSRI), citalopram (Citaxin, 20 mg, Bausch Health Ireland Limited, Dublin, Ireland), was used. Both the stimulated and sham CIT groups constituted positive controls for the potential anxiolytic effects of GOS, as CIT is commonly used in patients with depression and anxiety disorders [38]. The CIT solution was prepared once every three days by adding crushed tablets to water (3 mg of the drug was used per 30 mL of water) in order to obtain, as accurately as possible, the most commonly used dose (10 mg/kg bw/day) in animal studies [119]. Considering faster metabolism in small animals, the dosage of citalopram at 10 mg/kg/day (3 mg/day for a rat weighing about 300 g) correspond to a daily dose of 50 mg in humans, which is close to the highest dosage in the range for depression treatment [120].

### 4.5. Long-Term Electrical Stimulation of the CeA in Actometers

The electrical stimulation of the CeA and sham stimulation was performed daily for 14 consecutive days in the behavioural room between 8.00 and 12.00 a.m. During one session, three rats were stimulated in separate actometers, including two individuals subjected to electrical stimulation and one individual subjected to sham stimulation (identical experimental procedure but with the current switched off). The animals were removed from the housing cage, and the male amphenol connectors visible on the top of the implant were connected to compatible cable pins, the other end of which was plugged into the appropriate socket of the stimulator (215/I, Hugo Sachs Elektronik D7 806 March-Hugstetten, Germany). For habituation to the experimental conditions, the animals were allowed to explore the test box for 5 min. A single CeA stimulation session included 30 cycles, each consisting of 30 s of electric current stimulation followed by a 20 s rest period (current switched off). During stimulation, rectangular pulses were used, with the width of a single pulse of 0.1 ms and a constant frequency of 50 Hz. The initial current intensity was selected individually for each subject during the screening procedure (40–80 μA). In the absence of the expected behavioural response during three consecutive cycles, the current intensity was increased by 10 μA. At the end of each session, the current intensity at which CeA stimulation began on the next day was determined, and this value was gradually increased from session to session to avoid tissue accommodation. The maximum current intensity obtained during the last few days of stimulation reached approximately 200 μA.

### 4.6. Locomotor Activity

The animals were stimulated in actometers (Opto-Varimex Minor—Columbus, OH, USA), which allowed for the measurement of the rats’ motor activity during the procedure. After the handling procedure, the first measurement of motor activity was performed according to a method described by Dunacka et al. [121], with modifications. The rats were placed individually in the actometers for 30 min (8.00–12.00 a.m.), which constituted a new environment for exploration. During subsequent measurements, the conditions, the test apparatus and the procedure were known to the animals. The actometers were washed with an alcohol solution with detergent before each stimulation session to eliminate the influence of foreign odour traces. The test apparatus was a Plexiglas box (43 cm long, 43 cm wide, 20 cm high) with built-in sets of photocells (3 × 15 cells) emitting infrared beams that scan the area at a frequency of 150/min in two planes: horizontal (photocells placed 25 mm above the floor surface) and vertical (photocells placed on two parallel walls of the box, 175 mm above the floor surface). Based on the number of infrared beam obscurations caused by the animal’s movement, the digital counter generated the total number of movements recorded in each minute of measurement, classifying them as horizontal, vertical and ambulatory (e.g., grooming, sniffing and flinching) movements. Locomotor activity (horizontal, vertical and ambulatory) was assessed at 3 time points: (1) initially (basal locomotor activity), whereby a single session was performed 21 days before the first electrical stimulation of the CeA; (2) after 7 days of stimulation; and (3) after 14 days of stimulation, whereby the average of the results from the measurements performed during 1–7 and 8–14 days of stimulation, respectively, was determined.

### 4.7. The Anxiety Level Assessment

Anxiety behaviour in all groups of animals was measured immediately after the last session of long-term electrical stimulation of the CeA (14th day of stimulation and 21st day of supplementation). The elevated plus maze (EPM) test was used in this study, and it is the most commonly used anxiety test, and it belongs to the passive and active avoidance tests (e.g., [122]). The maze consisted of four narrow black arms (10 cm wide), which were placed crosswise, creating a “+” sign. Two opposite arms with side walls were closed arms of the maze (50 × 10 × 40 cm). The remaining two uncovered arms of the same dimensions were open arms, and they were connected to the closed arms via a central square (10 × 10 cm) located in the centre of the apparatus. Additionally, all arms of the maze were located at a height of 70 cm from the floor surface, which increased the aversive features of the test. The test was performed between 8.30 and 14.00 in an acoustically isolated room for behavioural studies with low light intensity. The animal was placed in the central square of the maze, with its snout facing one of the closed arms. The anxiety behaviour for each rat was measured for 10 min, and the test was recorded and analysed with the EthoVision XT 10 software (Noldus, Wageningen, the Netherlands) based on parameters such as frequency in closed and open arms and in the central square, the number of transitions between the central square and closed/open arms and the time spent on moving or not moving. After the measurement was completed and the animal was placed in the housing cage, the apparatus was thoroughly cleaned with a solution of detergent and alcohol to eliminate odour traces.

### 4.8. Euthanasia and Blood Sample Collection

One hour after the last behavioural test, each animal received a lethal dose of pentobarbital (Euthasol vet, 140 mg/kg bw, intraperitoneal injection, Produlab Pharma B.V., Raamsdonksveer, the Netherlands,). When the animal showed no reflex reactions, blood collection via cardiac puncture was initiated. Approximately 4 mL of blood was collected in 5 mL syringes connected to a needle rinsed in heparin (*Heparinum natricum* WZF, 5000 IU/mL, Polfa S.A. Warszawa, Poland). The needle was then removed, and the contents of the syringe were emptied into chilled glass centrifuge tubes containing 20 μL of heparin. The contents of the tube were gently mixed, and 250 μL of whole blood was collected in a labelled Eppendorf tube for haematological analysis. The tubes with the remaining blood were placed in a refrigerator until further procedures.

### 4.9. Haematological Analysis

The parameters of the white blood cell, red blood cell and platelet systems were determined in an automatic haematology analyser (ABX Micros ES 60, Horiba Medical, Loos, France). A total of 250 μL of whole blood in an Eppendorf tube was gently mixed by inverting the tube several times; then, the analyser needle was placed in it, which automatically collected 10 μL of blood and performed the analysis. The following haematological parameters were determined for each rat: the number and percentage of white blood cells (WBCs), including lymphocytes (LYMs), monocytes (MONs) and granulocytes (GRAs); red blood cell (RBC) count; haemoglobin (HGB) concentration; haematocrit (HCT); mean corpuscular haemoglobin concentration (MCHC); mean corpuscular haemoglobin (MCH); mean corpuscular volume (MCV); platelet (PLT) count; plateletcrit (PCT); and mean platelet volume (MPV).

### 4.10. Measurement of the Plasma Immunological Marker Concentration

Glass tubes with whole blood stored in a refrigerator were placed in a laboratory centrifuge (Jouan CR 3-22, Château-Gontier, France) and centrifuged for 10 min at 3000 rpm at 4 °C. Then, the plasma from each rat was pipetted into labelled Eppendorf microtubes, 200 μL to each tube. The prepared plasma was placed in a −70 °C low-temperature freezer and stored until further analysis.

The concentration of the selected immunological markers in plasma, namely tumour necrosis factor alpha (TNF-α) and interleukin 10 (IL-10), was determined using the immunoenzymatic ELISA method using the following commercial kits: the Rat TNF-alpha ELISA Kit (#ERA56RB, Invitrogen, ThermoFisher Scientific, Waltham, MA, USA) and the Rat IL-10 ELISA Kit (#ERA23RB, Invitrogen, ThermoFisher Scientific, Waltham, MA, USA) in accordance with the manufacturer’s instructions. The absorbance was read in a Synergy H1 microplate reader (BioTek, Winooski, VT, USA) at a wavelength of 450 nm at room temperature (21–25 °C). The standard curve was made based on the absorbance of the included standard solutions with known concentrations of the tested markers. Standard solutions and tested samples were applied to the plate in duplicate, and the average of the two readings was used for calculations. Based on the standard curve, the plasma concentration of TNF-α and IL-10 of each rat was calculated using the reader software (Gen5, BioTek, Winooski, VT, USA).

### 4.11. Histological Verification of Electrode Implantation

After euthanasia and blood collection, animals were subjected to brain tissue fixation via transcardial (via the left ventricle) perfusion with 200 mL of 0.9% saline followed by 200 mL of 4% paraformaldehyde (PFA) in 0.1 M phosphate-buffered saline (PBS). The isolated brains were placed in a container with 4% PFA and stored in a refrigerator (4 °C) for 24 h and then transferred to containers with a 30% sucrose solution in PBS at 4 °C for 48 h (cryoprotection). Coronal brain sections (20 μm) were cut using a cryostat (CM 786 1850, Leica Biosystems, Nussloch, Germany). For histological verification, 2 sections (every 3rd and 4th section) were selected from the same anteroposterior cross-sections for each rat: 1.8–2.4 mm posterior to bregma. Nissl staining of brain sections was used to identify the placement of stimulating electrodes in the CeA according to Jerzemowska et al. [110]. The sections stained with cresyl violet were mounted on microscope slides and were analysed with a microscope coupled with a camera (Stemi 508 + Axiocam 105 colour, Zeiss, Oberkochen, Germany). The borders of the brain structures were determined based on the rat brain atlas [39].

### 4.12. Gut Microbiota Analysis

During perfusion, the rat’s colon wall was incised with sterile scissors, and a sample of the intestinal content was collected and placed in labelled and weighed Eppendorf tubes containing 1 mL of saline (0.9% NaCl) and 500 μL of glycerol. The tubes were briefly vortexed, weighed, and immediately placed in a low-temperature freezer (−70 °C).

The analysis of the quantitative and qualitative composition of the intestinal microbiota was performed in cooperation with the Division of Medical Microbiology, Department of Microbiology, Faculty of Medicine, Medical University of Gdańsk. Serial dilutions of the intestinal content samples were inoculated onto a blood medium (Graso Biotech, Starogard Gdański, Poland) and a medium enabling the selective growth of *Bifidobacterium* bacteria (bifidus selective medium, BSM) (Merck Life Science, Poznań, Poland) to perform the quantitative analysis. The cultures were incubated for 48 h at 37 °C in anaerobic conditions. Based on the grown colonies, the number of colony-forming units (CFUs) in 1 mL of the sample (CFU/mL) was estimated for each colony morphology, and the bacteria species were identified. The qualitative analysis was performed using the Matrix-Assisted Laser Desorption/Ionisation—Time-Of-Flight (MALDI-TOF) mass spectrometry method, which is based on the detection of the presence of proteins unique to the bacteria species and comparing the obtained results with the database. In the case of qualitative analysis on a blood medium, an additional multiplication procedure was performed by adding 100 μL of intestinal content samples to 4 mL of a TSB (casein–soy broth) medium and incubating the mixture for 48 h under anaerobic conditions. Then, serial dilutions of the culture were made, and the samples were plated on a blood medium. The colonies that grew after 48 h of incubation were identified using the MALDI-TOF method.

### 4.13. Data Analysis

Statistical analysis was performed in Statistica 12 (Statsoft, Kraków, Poland). After checking the compliance with the normal distribution and the assumptions of the analysis of variance, the following analyses were performed: three-way ANOVA for locomotor activity with stimulation (stimulated_sham) and supplementation (GOS_CIT_water) between-subjects factors and the time points (basal_after 7 days_after 14 days of stimulation) as a within-subject factor and two-way ANOVA for other parameters (stimulation and supplementation as factors) followed by Tukey’s post hoc test. Effect sizes were measured using η_p_^2^. Data were expressed as the mean ± standard error of the mean ($\bar{x}$ ± SEM). The level of significance was set at *p* < 0.05. The correlation between the mean GOS or CIT solution intake and the frequency in the open arms in the EPM test was performed using Pearson’s correlation coefficient.

## 5. Conclusions

Our results indicated that GOS have high therapeutic potential in limiting the adverse changes induced by electrical stimulation of the CeA, which may lead to further research using animal models, as well as clinical trials verifying the efficacy of these prebiotics in anxiety disorders, depression or PTSD, at least as a supplement to conventional therapy. We demonstrated that GOS supplementation showed even greater therapeutic potential than citalopram, most likely due to more effective prevention of gut microbiome dysbiosis and a lower level of risk of side effects with long-term use. In addition, due to the high level of safety and multifaceted health benefits, the obtained results may also contribute to further research on the more widespread use of GOS to prevent the development of anxiety- and stress-related disorders.

## Figures and Tables

**Table 1:** Locomotor activity assessed in actometers at three time points (F and *p* values of ANOVA analysis and effect sizes measured using η_p_^2^).

Factors	df	HOR			VER			AMB		
		F	*p*	ES	F	*p*	ES	F	*p*	ES
Electrical stimulation of the CeA (Stim_Sham)	1, 126	**104.46**	<0.001	0.45	**124.81**	<0.001	0.72	**178.57**	<0.001	0.82
Supplementation (GOS_CIT_water)	2, 126	**30.69**	0.002	0.33	**26.11**	<0.001	0.29	**68.22**	<0.001	0.52
Time point (basal_7 days after_14 days after stimulation)	2, 126	**59.93**	<0.001	0.49	**132.80**	<0.001	0.68	**98.44**	<0.001	0.61
Interactions:										
(1) Stimulation × supplem.	2, 126	**37.92**	0.002	0.38	**23.73**	0.004	0.27	**61.10**	<0.001	0.49
(2) Stimulation × Time point	2, 126	**23.84**	0.003	0.27	**91.95**	<0.001	0.59	**156.83**	<0.001	0.71
(3) Supplem. × Time point	4, 126	**12.21**	0.007	0.28	**21.04**	0.003	0.40	**57.81**	<0.001	0.65
(4) Stimulation × supplem. × Time point	4, 126	**7.54**	0.013	0.19	**14.29**	0.007	0.31	**49.61**	<0.001	0.61

Explanations: df—degrees of freedom; Stim—electrical stimulation of the CeA; Sham—sham stimulation; GOS—galactooligosaccharide supplementation; Supplem.—supplementation; CIT—citalopram administration; ES—effect size. Statistically significant (*p* < 0.05) results are marked in bold.

**Table 2:** Anxiety level assessed in the elevated plus maze (F and *p* values of ANOVA analysis and effect sizes measured using η_p_^2^).

		Factors											
		Electrical Stimulation of the CeA (Stim_Sham)				Supplementation (GOS_CIT_Water)				Interaction (Stimulation × Supplementation)			
Variables		df	F	*p*	ES	df	F	*p*	ES	df	F	*p*	ES
Frequency	Central square	1, 42	0.19	0.664	0.00	2, 42	**8.32**	0.001	0.28	2, 42	1.12	0.337	0.05
	Closed arms	1, 42	1.29	0.262	0.03	2, 42	2.16	0.128	0.09	2, 42	0.47	0.625	0.02
	Open arms	1, 42	2.38	0.130	0.05	2, 42	**25.39**	<0.001	0.55	2, 42	**4.79**	0.013	0.19
Number of transitions	Central square >> Closed arms	1, 42	0.12	0.730	0.00	2, 42	**4.45**	0.018	0.17	2, 42	1.28	0.288	0.06
	Central square >> Open arms	1, 42	0.01	0.937	0.00	2, 42	**18.38**	<0.001	0.47	2, 42	**7.15**	0.002	0.25
Time spent	Moving	1, 42	0.10	0.749	0.00	2, 42	**4.45**	0.018	0.17	2, 42	**6.67**	0.003	0.24
	Not moving	1, 42	0.11	0.749	0.00	2, 42	**4.50**	0.018	0.17	2, 42	**6.47**	0.003	0.24

Explanations: df—degrees of freedom; Stim—electrical stimulation of the CeA; Sham—sham stimulation; GOS—galactooligosaccharide supplementation; CIT—citalopram administration; ES—effect size. Statistically significant (*p* < 0.05) results are marked in bold.

**Table 3:** Pearson’s correlation coefficient (r values) between the mean GOS or CIT solution intake and the frequency in the open arms in the EPM test.

Groups	Mean GOS or CIT Solutions Intake	Frequency in the Open Arms	r Values
ShamGOS	29.00	17.13	0.66
ShamCIT	28.21	13.88	0.65
StimGOS	28.88	17.88	0.84 *
StimCIT	27.88	14.63	0.83 *

* *p* < 0.05 significant correlation.

**Table 4:** Haematological parameters (F and *p* values of ANOVA analysis and effect sizes measured using η_p_^2^).

	Factors											
	Electrical Stimulation of the CeA (Stim_Sham)				Supplementation (GOS_CIT_Water)				Interaction (Stimulation × Supplementation)			
Variables	df	F	*p*	ES	df	F	*p*	ES	df	F	*p*	ES
WBC [10^3^/mm^3^]	1, 42	**9.64**	0.003	0.19	2, 42	**38.02**	<0.001	0.64	2, 42	**27.50**	<0.001	0.57
LYM [10^3^/mm^3^]	1, 42	**9.18**	0.004	0.18	2, 42	**22.19**	<0.001	0.51	2, 42	**14.45**	<0.001	0.41
MON [10^3^/mm^3^]	1, 42	0.01	0.919	0.00	2, 42	**8.25**	<0.001	0.28	2, 42	**5.25**	0.009	0.20
GRA [10^3^/mm^3^]	1, 42	0.11	0.738	0.00	2, 42	**5.69**	0.006	0.21	2, 42	**4.41**	0.018	0.17
LYM [%]	1, 42	2.89	0.096	0.06	2, 42	0.26	0.770	0.01	2, 42	0.56	0.557	0.03
MON [%]	1, 42	2.59	0.115	0.06	2, 42	0.73	0.490	0.03	2, 42	0.38	0.686	0.02
GRA [%]	1, 42	3.58	0.065	0.08	2, 42	2.71	0.078	0.11	2, 42	3.04	0.058	0.13
RBC [10^6^/mm^3^]	1, 42	**11.37**	0.002	0.21	2, 42	**5.86**	0.006	0.22	2, 42	**9.12**	<0.001	0.30
HGB [g/dL]	1, 42	**6.00**	0.018	0.12	2, 42	**3.28**	0.048	0.13	2, 42	**4.07**	0.024	0.16
HCT [%]	1, 42	**23.51**	<0.001	0.36	2, 42	**10.28**	<0.001	0.33	2, 42	**15.61**	<0.001	0.43
MCV [µm^3^]	1, 42	**7.16**	0.010	0.15	2, 42	3.20	0.051	0.13	2, 42	**5.39**	0.008	0.20
MCH [pg]	1, 42	0.10	0.750	0.00	2, 42	0.79	0.460	0.04	2, 42	0.05	0.953	0.00
MCHC [g/dL]	1, 42	2.63	0.112	0.06	2, 42	1.07	0.353	0.05	2, 42	1.85	0.170	0.08
PLT [10^3^/mm^3^]	1, 42	2.22	0.144	0.05	2, 42	**4.19**	0.022	0.17	2, 42	1.78	0.181	0.08
MPV [µm^3^]	1, 42	0.72	0.400	0.02	2, 42	**7.20**	0.002	0.26	2, 42	0.19	0.825	0.01
PCT [%]	1, 42	**5.19**	0.028	0.11	2, 42	**3.70**	0.033	0.15	2, 42	**5.70**	0.006	0.21

Explanations: df—degrees of freedom, Stim—electrical stimulation of the CeA, Sham—sham stimulation, GOS—galactooligosaccharide supplementation, CIT—citalopram administration, ES—effect size, WBC—white blood cell, LYM—lymphocyte, MON—monocyte, GRA—granulocyte, RBC—red blood cell, HGB—haemoglobin concentration, HCT—haematocrit, MCV—mean corpuscular volume, MCH—mean corpuscular haemoglobin, MCHC—mean corpuscular haemoglobin concentration, PLT—platelet, MPV—mean platelet volume and PCT—plateletcrit. Statistically significant (*p* < 0.05) results are marked in bold.

**Table 5:** Plasma immunological markers (F and *p* values of ANOVA analysis and effect sizes measured using η_p_^2^).

	Factors											
	Electrical Stimulation of the CeA (Stim_Sham)				Supplementation (GOS_CIT_Water)				Interaction (Stimulation × Supplementation)			
Variables	df	F	*p*	ES	df	F	*p*	ES	df	F	*p*	ES
TNF-α [pg/mL]	1, 42	**63.86**	<0.001	0.60	2, 42	**5.68**	0.006	0.21	2, 42	**7.60**	0.002	0.27
IL-10 [pg/mL]	1, 42	**9.38**	0.004	0.18	2, 42	**50.03**	<0.001	0.70	2, 42	**22.83**	<0.001	0.52

Explanations: df—degrees of freedom; Stim—electrical stimulation of the CeA; Sham—sham stimulation; GOS—galactooligosaccharide supplementation; CIT—citalopram administration; ES—effect size, TNF-α—tumour necrosis factor alpha, IL-10—interleukin 10. Statistically significant (*p* < 0.05) results are marked in bold.

**Table 6:** The number of species most abundant in the rats’ gut microbiota (F and *p* values of ANOVA analysis and effect sizes measured using η_p_^2^).

	Factors											
	Electrical Stimulation of the CeA (Stim_Sham)				Supplementation (GOS_CIT_Water)				Interaction (Stimulation × Supplementation)			
Variables [% CFU]	df	F	*p*	ES	df	F	*p*	ES	df	F	*p*	ES
*Enterococcus* *gallinarum*	1, 23	0.20	0.661	0.01	2, 23	1.47	0.250	0.12	2, 23	1.14	0.338	0.09
*Enterococcus* *faecalis*	1, 23	1.43	0.244	0.06	2, 23	**8.63**	0.002	0.44	2, 23	0.99	0.389	0.08
*Bifidobacterium* *animalis*	1, 23	**17.73**	<0.001	0.44	2, 23	**11.89**	<0.001	0.51	2, 23	**3.32**	0.047	0.22
*Enterococcus* *casseliflavus*	1, 23	**38.03**	<0.001	0.62	2, 23	**36.57**	<0.001	0.76	2, 23	**36.57**	<0.001	0.76
*Ligilactobacillus* *murinus*	1, 23	**4.43**	0.046	0.16	2, 23	**3.27**	0.048	0.22	2, 23	**4.00**	0.032	0.26
*Lactobacillus* *johnsonii*	1, 23	0.84	0.368	0.04	2, 23	0.04	0.962	0.00	2, 23	0.74	0.490	0.06
*Limosilactobacillus* *reuteri*	1, 23	**11.44**	0.002	0.33	2, 23	**4.37**	0.024	0.28	2, 23	**4.20**	0.028	0.27

Explanations: df—degrees of freedom; Stim—electrical stimulation of the CeA; Sham—sham stimulation; GOS—galactooligosaccharide supplementation; CIT—citalopram administration; ES—effect size; CFU—colony-forming unit. Statistically significant (*p* < 0.05) results are marked in bold.

## Data Availability

The raw data supporting the conclusions of this article will be made available by the authors on request.

## Abbreviations

AMB	ambulatory movement
Amg	amygdala
BLA	basolateral amygdala
BNST	bed nucleus of stria terminalis
BSM	bifidus selective medium
CeA	central nucleus of the amygdala
CFU	colony-forming unit
CIT	citalopram
CNS	central nervous system
EPM	elevated plus maze
FOS	fructooligosaccharide
GABA	gamma-aminobutyric acid
GOS	galactooligosaccharide
GRA	granulocyte
HCT	haematocrit
HGB	haemoglobin concentration
HOR	horizontal movements
HPA	hypothalamic–pituitary–adrenal axis
IL	interleukin
LPS	lipopolysaccharide
LYM	lymphocyte
MCH	mean corpuscular haemoglobin
MCHC	mean corpuscular haemoglobin concentration
MCV	mean corpuscular volume
MON	monocyte
MPV	mean platelet volume
PCT	plateletcrit
PLT	platelet
PTSD	post-traumatic stress disorder
RBC	red blood cell
SSR	selective serotonin reuptake inhibitor
TNF	tumour necrosis factor
VER	vertical movement
WBC	white blood cell
